# High Mobility Group Protein B1 Promotes Interferon Regulatory Factor 1 SUMOylation to Prime Trained Immunity of Circulating Monocytes and Aggravate the Progressive Synovial Inflammation in Knee Osteoarthritis

**DOI:** 10.34133/research.1243

**Published:** 2026-05-14

**Authors:** Lishi Jie, Jun Mao, Li Zhang, Houyu Fu, Xiaochen Li, Taiyang Liao, Yibao Wei, Deren Liu, Jiaojiao Du, Peng Wu, Songjiang Yin, Nongshan Zhang, Meng Cao, Peimin Wang

**Affiliations:** ^1^ Department of Orthopaedics and Traumatology, Affiliated Hospital of Nanjing University of Chinese Medicine, Jiangsu Provincial Hospital of Traditional Chinese Medicine, Nanjing 210023, Jiangsu, China.; ^2^ Key Laboratory for Metabolic Diseases in Chinese Medicine, First College of Clinical Medicine, Nanjing University of Chinese Medicine, Jiangsu Provincial Hospital of Traditional Chinese Medicine, Nanjing 210023, Jiangsu, China.; ^3^ Jiangsu Provincial Engineering Research Center of TCM External Medication Development and Application, Nanjing 210023, Jiangsu, China.; ^4^ Department of Traditional Chinese Orthopedics, Zhongda Hospital, Southeast University, Nanjing 210023, Jiangsu, China.; ^5^ Jiangsu Provincial Medical Innovation Center, Affiliated Hospital of Integrated Traditional Chinese and Western Medicine, Nanjing University of Chinese Medicine, Nanjing 210023, Jiangsu, China.

## Abstract

Knee osteoarthritis (KOA) is clinically characterized by recurrent and progressively worsening episodes; however, its underlying pathological mechanisms remain incompletely understood. Phenotypic changes and the specific migration of circulating monocytes may be key factors contributing to the recurrence and progressive exacerbation of synovial inflammation in KOA. In this study, we report a specific subtype of circulating monocytes in KOA that highly express interleukin 1A (IL1A), IL1R1, tumor necrosis factor, CXCL12, CXCL3, CXCL2, CCL20, and G0S2. These monocytes exhibit a trained immune phenotype, and their CXCR4-dependent migration to the synovium exacerbates synovial inflammation. HMGB1 (high mobility group protein B1) primes the trained immunity of this subtype, resulting in increased chromatin accessibility, and the transcriptional storage of multiple proinflammatory factors enables them to exhibit a more positive inflammatory response when restimulated by HMGB1. In addition, we find that MyD88, downstream of HMGB1, recruits cytoplasmic interferon regulatory factor 1 to the nucleus and then stabilizes interferon regulatory factor 1 in chromatin through SUMOylation of tripartite motif containing 28 to exert transcriptional activity and epigenetic enhancement of proinflammatory factor expression. Finally, we found that KOA inflammation was effectively attenuated by blocking HMGB1. Taken together, this study reveals the mechanism by which trained immunity of circulating monocytes promotes the progression of synovial inflammation, supporting a future therapeutic approach in the management of KOA.

## Introduction

In the “wear and tear” theory, knee osteoarthritis (KOA) is generally considered to be a degenerative disease characterized primarily by cartilage damage, while inflammation, both locally in the joint and systemic, is nowadays considered among the mechanisms involved [1–5]. Synovitis, mainly inflammation of the synovial lining layer, shows obvious flares and subsides in clinical symptoms and is closely related to progressive aggravation of the disease and the increase of pain sensitivity [2,6–8]. The lining layer is mainly composed of fibroblast-like synoviocytes (FLSs) and macrophages, which respond to stimuli from a variety of damage-associated molecular patterns (DAMPs) and release classical proinflammatory factors such as interleukin-1β (IL-1β), IL-6, and tumor necrosis factor-α (TNF-α) [8,9]. Our previous studies supported that the activation of nucleotide-binding oligomerization domain-like receptor thermal protein domain associated protein 3 inflammasome in FLSs or macrophages of KOA, caused the massive release of high mobility group protein B1 (HMGB1), IL-1β, and IL-18 and promoted the progression of synovial inflammation [10,11].

Targeting of DAMPs in immune cells is an effective method of altering their behavior and the associated inflammatory responses of KOA [10,12,13]. However, unlike adaptive immunity, this innate immunity lacks the corresponding memory after the stimulus is removed; on the contrary, even brief microbial stimulation can lead to long-term changes in immune cells, leading to being more responsive when challenged again by the same microbe or stimulus [14,15]. Within this context, accumulating evidence has challenged the dogma that only lymphoid cells are privileged with immunological memory; notably, FLSs and macrophages in synovium appear to have immunological memory, in consequence sensitizing tissue for inflammation [16–18]. At the molecular level, this concept of “innate immune memory” is named “trained immunity” and recognized as the result of well-characterized metabolic and epigenetic changes leading to chromatin modifications and altered susceptibility for transcription of inflammation-associated genes [19,20].

As one of the most important immune cells in innate immunity, macrophages of synovial membrane can be divided into local resident and cycling-derived, which are independent of each other [21]. Local resident macrophages do not depend on the supplement of circulation-derived macrophages [21,22]. This allows these 2 types to have different functions in the progression of synovial inflammation in KOA; locally resident macrophages are protective, while cycling-derived macrophages are destructive, in general [22–24]. Circulation-derived macrophages originate in the bone marrow, differentiate into circulating monocytes, and migrate to the synovial membrane [22–24]. Frontier research points out that IL-1-primed maladaptive trained immunity in bone marrow underlies inflammatory comorbidities. Experimental-periodontitis-related systemic inflammation in mice induced epigenetic rewiring and led to sustained enhancement of production of myeloid cells with increased inflammatory preparedness, which exhibited increased inflammatory responsiveness and disease severity when subjected to inflammatory arthritis [25]. Besides, Bacillus Calmette–Guérin-trained monocyte-derived macrophages showed osteogenic differentiation, up-regulated alkaline phosphatase activity, mineralization, and cytokine (TNF and IL-6) levels, cocultured with human mesenchymal stromal cells [26].

Accordingly, macrophage in both central immunity of bone marrow and peripheral immunity of circulation may undergo trained immunity. Notably, the activation of circulating monocytes has been identified as a risk factor for KOA, and metabolism-related risk can activate circulating monocytes prior to infiltration into the synovium and aggravate KOA via proinflammatory skewing of the synovial macrophage population [27]. It has been demonstrated in mouse models that Ly6C^+^ circulating monocytes, which resemble the human CD14^+^ population, are preferentially recruited to the inflamed joint and give rise to inflammatory macrophages [28]. Up-regulation of plasma CCL3 and CCL4 in KOA patients was associated with the radiographic severity of KOA and showed substantial ability to recruit CD14^+^CD16^−^ monocytes in transwell assays [21,28–30]. Furthermore, monocytes are activated prior to their entry into the synovium and show heterogeneity in KOA patients compared to healthy people [31,32]. This makes us particularly interested in exploring the specific subpopulation of circulating monocytes in KOA and discussing whether circulating monocytes may undergo trained immunity to participate in progressive synovial inflammation in KOA.

In this study, we extracted circulating monocytes from the peripheral blood of healthy volunteers and KOA patients and confirmed that KOA-derived circulating monocytes showed a trained phenotype when they were restimulated by inflammation proinflammatory factors. Then, we applied single-cell sequencing and assay for transposase-accessible chromatin (ATAC) sequencing to analyze the differences between the circulating monocytes from both groups of people. We identified a specific subpopulation of circulating monocytes that highly expressed *IL1A*, *IL1R1*, *TNF*, *CXCL12*, *CXCL3*, *CXCL2*, *CCL20*, and *G0S2*. These cells exhibit increased chromatin accessibility and possess transcriptional priming for various proinflammatory factors, including interferon (IFN) regulatory factors interferon regulatory factor 1 (*IRF1*) and *IRF3*. CXCR4 mediates the specific migration of this particular subgroup into the synovial tissue of KOA and releases more proinflammatory factors at the next stimulation. In addition, we identified HMGB1 as the DAMP that drives CXCR4^+^ monocytes to train immunity, and HMGB1 promoted the transfer of IRF1 from cytoplasm to nucleus via MyD88, followed by SUMOylation mediated by tripartite motif containing 28 (TRIM28). SUMOylated IRF1 stabilized in the nucleus enables monocytes to have a transcriptional reserve of proinflammatory factors and thus encode more inflammatory factors at the next stimulation. Our study provides a scientific explanation for the molecular mechanism of the progressive exacerbation of synovial inflammation in KOA and also provides a possibility for future treatment targeting the sensitivity of synovial inflammation in KOA.

## Results

### Single-cell transcriptomic analysis revealed the most pronounced inflammation-response-related signature among circulating monocytes

**Fig. 1.**
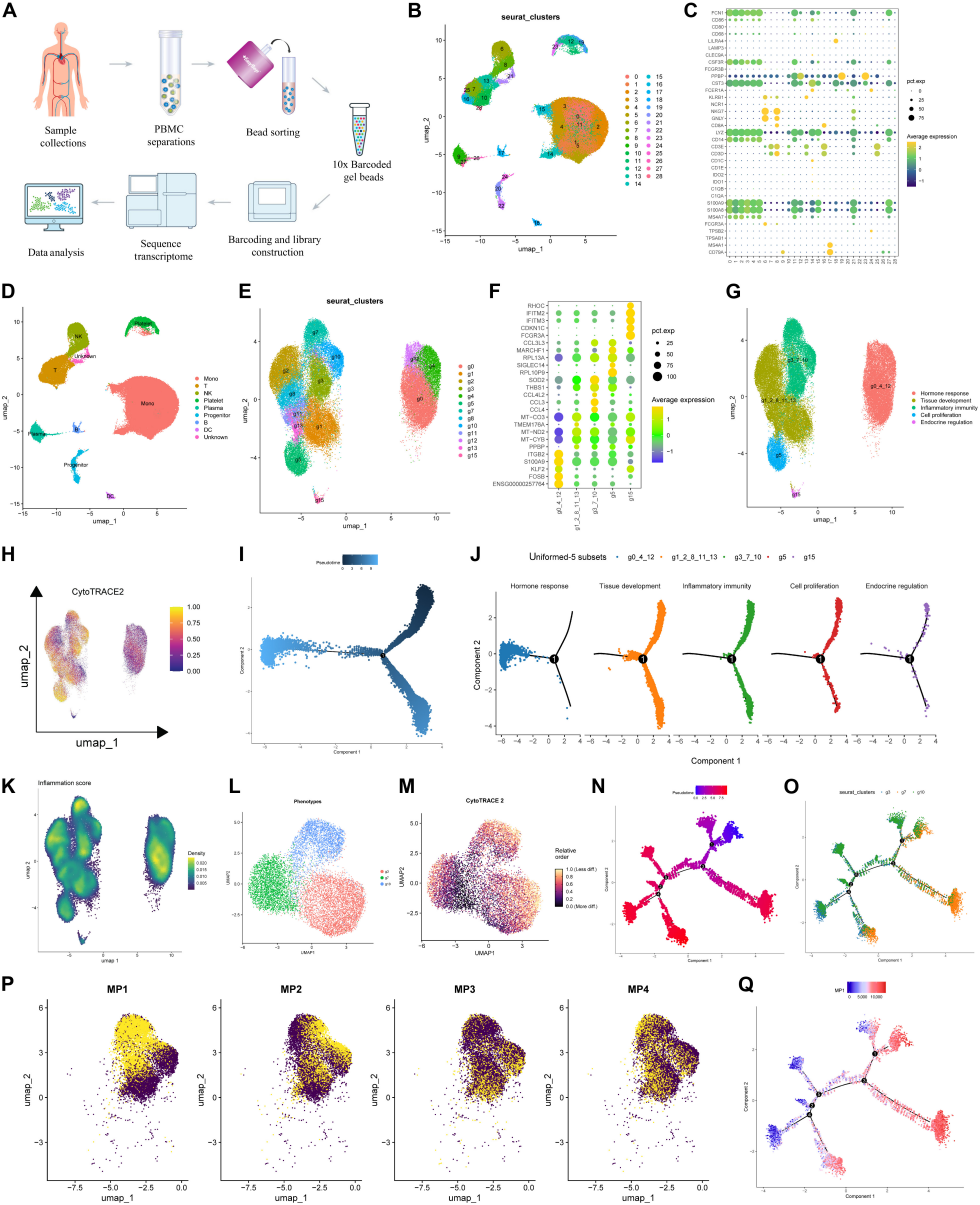
Single-cell transcriptomic analysis revealed the most pronounced inflammation-response-related signature among circulating monocytes. (A) Summary diagram of the process of collecting peripheral circulating cells from clinical samples for 10x Genomics single-cell sequencing. (B) Uniform manifold approximation and projection (UMAP) dimensionality reduction analysis was used to visualize the spatial distribution and classification of all cells. (C) Bubble plots were used to display the expression levels of marker genes in each cell type. (D) UMAP dimensionality reduction analysis was employed to annotate all cells as specific cell types. (E) After removing low-quality cell subpopulations, UMAP dimensionality reduction analysis was used to show the distribution of the remaining circulating monocyte subpopulations. (F) Bubble plots were used to display the expression levels of marker genes in each circulating monocyte subpopulation. (G) Functional clustering of circulating monocyte subpopulations was performed, resulting in 5 functional groups. (H) CytoTRACE2 score was used to assess the differentiation potential of circulating monocytes. (I) Pseudotime analysis was used to visualize the differentiation trajectory of all circulating monocytes. (J) Pseudotime analysis was used to visualize the differentiation trajectories of circulating monocytes in the 5 functional groups. (K) Inflammation score was used to evaluate the scores of circulating monocytes in the 5 functional groups. (L) UMAP was used to display the distribution of cells in the g3-7-10 functional groups. (M) The CytoTRACE2 score was projected onto the UMAP distribution of the g3-7-10 functional groups to show the differentiation potential of circulating monocytes. (N) Pseudotime analysis was used to display the differentiation trajectories of circulating monocytes in the g3-7-10 functional groups. (O) Circulating monocytes from the g3, g7, and g10 subgroups were projected onto the pseudotime differentiation trajectory. (P) The MP1, MP2, MP3, and MP4 gene sets were projected onto the UMAP distribution of the g3-7-10 functional groups. (Q) Projection of the MP1 gene set onto the differentiation trajectory of circulating monocytes in the g3-7-10 functional groups revealed a high degree of overlap with the g7 distribution.

To further study circulating monocytes-trained immunity of KOA, we conducted 10x Genomics single-cell RNA sequecing (RNA-seq) and analyzed magnetic beads-sorted circulating monocytes, defined by their surface markers as CD14^+^ CD16^−^ from the peripheral blood of 6 healthy donor (HD) and 6 KOA patients (Fig. 1A). Following quality control and data filtering, ~100,000 high-quality, deeply sequenced cells were obtained, and a total of 29 distinct clusters were identified. Each cluster was defined by comparing its expression profile to known lineage markers or canonical gene signatures (Fig. 1B and C). We verified that the transcriptomic profile of the sorted cells contained the core cell signature of B cells, conventional dendritic cells, monocytes, plasmacytoid dendritic cells, plasma cells, platelets, stem cells, and T cells, as previously defined [33,34]. Monocytes accounted for the vast majority of these cells and, after the removal of low-quality cells, could be further classified into 13 subpopulations (Fig. 1D and E). They could be clustered into 5 functional subsets based on their signature genes (Fig. 1F and G and Fig. S1A to C), as follows: The “Hormone Response” subset (g0-4-12) is characterized by high expression of *ITGB2*, *S100A9*, and *FOSB*, and the differential features were mainly enriched “androgen response” and “estrogen response”, which may reflect the sex-specific features of KOA. The “Tissue Development” subset (g1-2-8-11-13) predominantly expresses *MT-CO3*, *MT-ND2*, and *MT-CYB*, and its differential features are enriched in pathways including “hedgehog signaling”, “apical junction”, and “epithelial mesenchymal transition”, potentially relating to synovial fibrosis in KOA. The “Inflammatory Immunity” subset (g3-7-10) is marked by high expression of *SOD2*, *CCL3*, and *CCL4*, and its distinct characteristics are mainly associated with enrichment in “inflammatory response”, “TNF-α signaling via NF-κB”, and “complement”, which may reflect the inflammatory immune processes in KOA. The “Cell Proliferation” subset (g5) mainly expresses *MARCHF1*, *RPL13A*, and *PL10P9* and is enriched in “PI3K-AKT-MTOR signaling”, “TGF-beta signaling” pathways, indicating possible cellular activation in KOA. The “Endocrine Regulation” subset (g15) shows high expression of *IFITM2*, *IFITM3*, *CDKN1C*, and *FCGR3A*, with its distinct features enriched in “bile acid metabolism”, “pancreas beta cells”, and “glycolysis”, which may suggest a metabolic component in KOA.

We further assessed the cellular stemness of each subset (Fig. 1H) and found that the “Hormone Response” subset exhibited lower differentiation potential. Pseudotime analysis confirmed similar findings, except for the “Hormone Response” subset. The remaining 4 subsets exhibited continuous cellular differentiation, indicating that they may dynamically adapt their phenotypes during KOA progression (Fig. 1I and J). Given our particular interest in trained immunity in monocytes, we assessed the inflammatory scores of each subset. As expected, the “Inflammatory Immunity” subset exhibited the highest inflammatory score among all subsets (Fig. 1K). We subsequently characterized the phenotypes of g3, g7, and g10 and analyzed their respective cellular stemness (Fig. 1L and M). Consistent with the results of the pseudotime analysis, no significant differences were observed among g3, g7, and g10, all of which demonstrated a robust and continuous differentiation capacity (Fig. 1N and O). To explore expression heterogeneity in the “Inflammatory Immunity” subset, we first defined intra-subset expression programs that consist of coexpressed genes in each cell using nonnegative matrix factorization. These expression programs represented gene modules that were highly expressed by g3, g7, and g10 (Fig. S1D). In total, we classified 4 metaprograms (MPs) shared by multiple monocyte (Fig. 1P) and selected the top 15 genes to characterize each MP module. MP1 was characterized by expression of genes such as *IL1A*, *IL1R1*, *TNF*, and *CXCL12*. MP2 consisted of genes (i.e., *RUNX3* and *ISG15*), while MP3 was enriched for genes such as *TGIF1* and *RHOB*. Besides, MP4 consisted of genes such as *LUCAT1*, *TRIB1*, and *S1PR3*. Notably, MP1 was perfectly mapped onto the pseudotime analysis trajectory and almost completely overlapped with g7 (Fig. 1Q), suggesting that it may represent the circulating monocyte subset with the most pronounced inflammation-response-related signature during KOA progression.

### Characterization of circulating monocytes subpopulation g7 in KOA

**Fig. 2.**
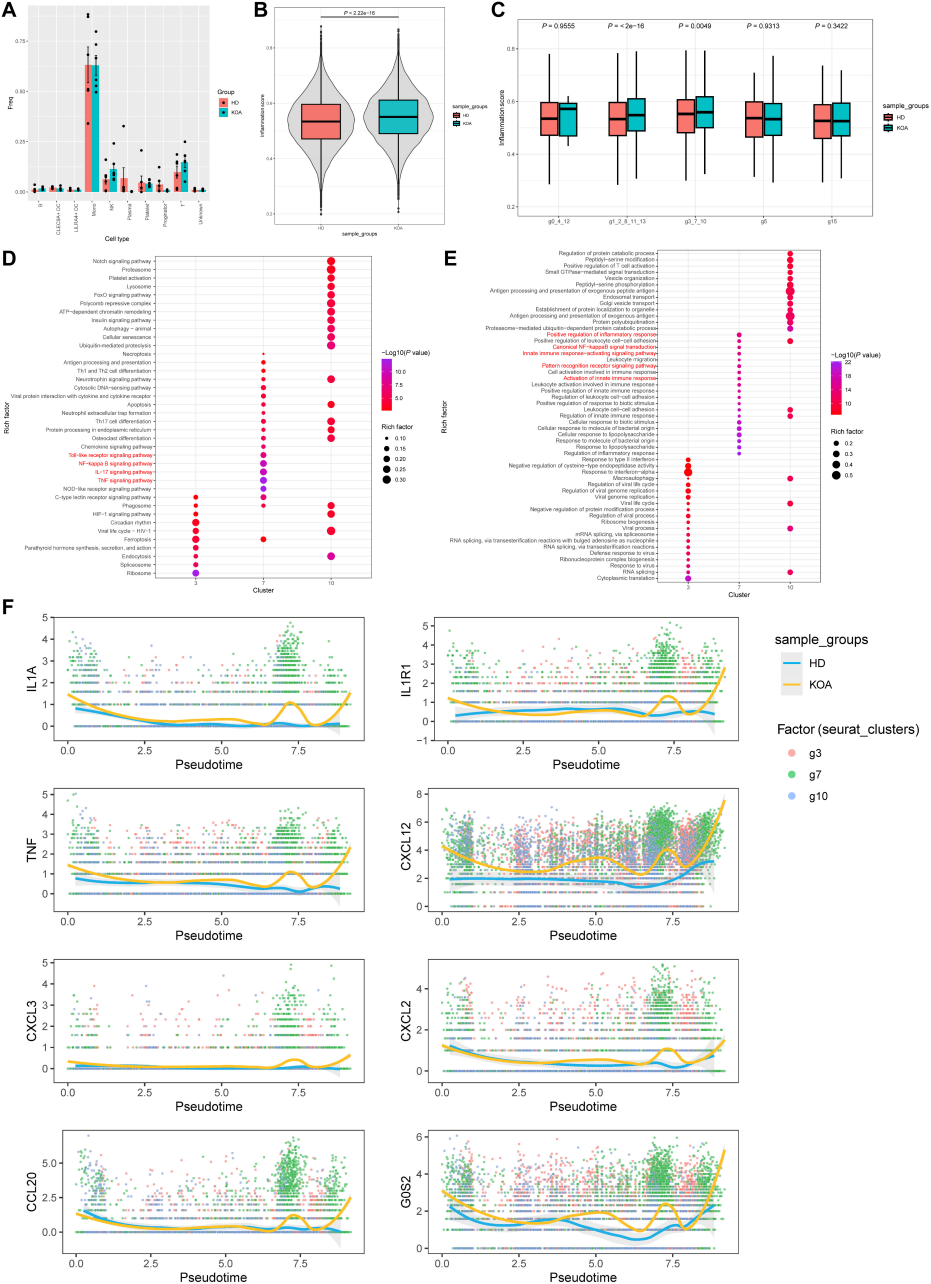
Characterization of circulating monocytes subpopulation g7 in knee osteoarthritis (KOA). (A) A heatmap shows the proportion of different cell types in the healthy donor (HD) group and the KOA group. (B) Circulating monocytes from the HD and KOA groups are evaluated based on the inflammation score. (C) The 5 functional subgroups of circulating monocytes in the HD and KOA groups are assessed based on the inflammation score. (D) Differentially expressed genes between the HD and KOA groups in the g3, g7, and g10 cell subpopulations are compared and subjected to enrichment analysis using the Kyoto Encyclopedia of Genes and Genomes (KEGG) database. (E) Enrichment analysis of differentially expressed genes between the HD and KOA groups in the g3, g7, and g10 cell subpopulations is also performed using the Gene Ontology (GO) database. (F) Genes including *IL1A*, *IL1R1*, *TNF*, *CXCL12*, *CXCL3*, *CXCL2*, *CCL20*, and *G0S2* from the MP1 gene cluster are mapped onto the differentiation trajectories of g3, g7, and g10, and the expression levels of these genes between the HD and KOA groups are presented. Significant differences in gene expression during the differentiation process are observed.

To better define g7, the highly inflammation-associated circulating monocyte population, and identify its differences between HD and KOA patients, we systematically investigated the differences in single-cell sequencing results between the 2 groups. In terms of quantity, the overall number of circulating monocytes did not differ between the 2 groups (Fig. 2A). However, the inflammatory score was significantly elevated in KOA patients (Fig. 2B) and demonstrated significant differences among the g3, g7, and g10 subsets (Fig. 2C). Differential expressed genes (DEGs) were mainly enriched in the “toll-like receptor signaling pathway”, “NF-κB signaling pathway”, “IL-17 signaling pathway”, and “TNF signaling pathway” (Fig. 2D, Kyoto Encyclopedia of Genes and Genomes [KEGG]) and enriched in the “positive regulation of inflammatory response”, “canonical NF-κB signal transduction”, “innate immune response-activating signaling pathway”, and “pattern recognition receptor signaling pathway” (Fig. 2E, Go). In addition, the differences in the MP1 gene module between HD and KOA were mainly enriched in the groups “Interleukin-1 signaling” and “TNFR-induced NF-κB pathway” (Fig. S2A, Reactome) and groups “Chemokine-cytokine interaction” and “IL-17 and TNF signaling pathway” (Fig. S2B, KEGG). Pseudotime analysis of *IL1A*, *IL1R1*, *TNF*, *CXCL12*, *CXCL3*, *CXCL2*, *CCL20*, and *G0S2* clearly distinguishes between HD and KOA and maps completely onto g7 (Fig. 2F and Fig. S2C). By contrast, pseudotime analysis of *KCNJ2*, *MAILR*, *MIR155HG*, *CCL3L3*, *CCL3*, *CCL4L2*, and *CCL4* does not clearly distinguish between the 2 groups (Fig. 2F and Fig. S2D). At this point, we characterized g7, a distinct, continuously differentiating inflammatory subpopulation, within circulating monocytes from KOA patients. This subpopulation is distinguished from other monocytes by its high expression of *IL1A*, *IL1R1*, *TNF*, *CXCL12*, *CXCL3*, *CXCL2*, *CCL20*, and *G0S2*.

### KOA-derived circulating monocytes undergo a trained phenotype

**Fig. 3.**
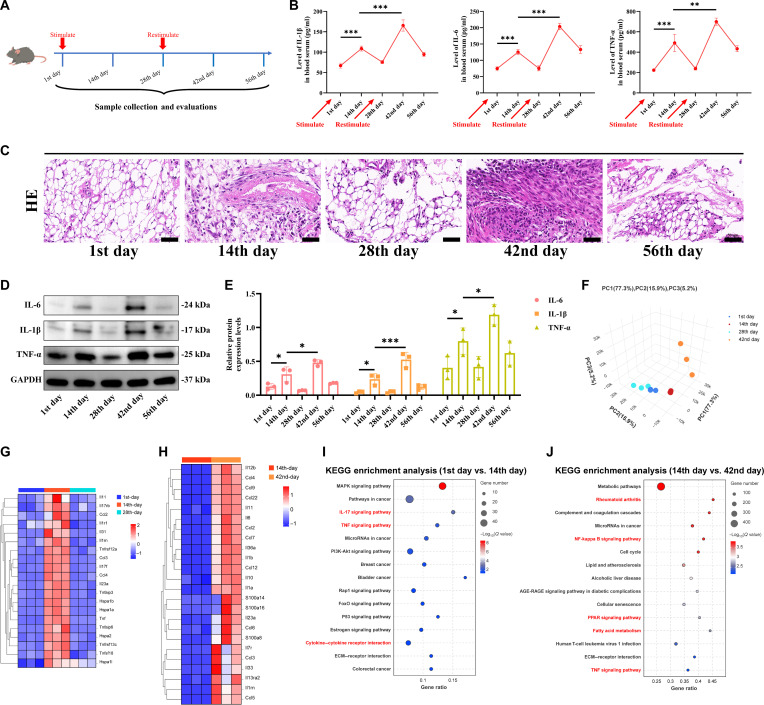
Knee osteoarthritis (KOA)-derived circulating monocytes undergo a trained phenotype. (A) Schematic diagram of the animal experimental procedure. (B) Detection of inflammatory factor levels in peripheral serum of mice at various time periods by enzyme-linked immunosorbent assay (ELISA) (*n* = 6). (C) Histopathological changes in mouse synovial tissue at various time periods using hematoxylin and eosin (HE) staining. (D) Detection of interleukin-1β (IL-1β), IL-6, and tumor necrosis factor-α (TNF-α) levels in peripheral circulating mononuclear cells of mice at various time periods using Western blot. (E) Statistical histogram of IL-1β, IL-6, and TNF-α in peripheral circulating monocytes of mice (*n* = 3). (F) Principal component analysis (PCA) of changes in transcript levels in peripheral circulating monocytes of mice at different periods of time. (G) Heatmap of differential genes among inflammatory factors, chemokines, and alertin family protein factors among 1st-day, 14th-day, 28th-day, and 42nd-day groups. (H) Heatmap of differentially expressed genes for inflammatory factors, chemokines, and alarmin family proteins among groups on day 14 and day 42. (I) Kyoto Encyclopedia of Genes and Genomes (KEGG) enrichment analysis of differential genes in the 1st-day and 14th-day groups. (J) KEGG enrichment analysis of differential genes in the 14th-day and 42nd-day groups. Statistical results are represented by mean ± standard error of the mean (mean ± SEM), *P < 0.05, **P < 0.01, ***P < 0.001.

In order to observe whether circulating monocytes in KOA undergo trained immunity, we first employed monoiodoacetic acid (MIA) articular injected mice to simulate the clinical progression of KOA synovial inflammation [35]. Previous studies reported that synovial inflammation in this KOA model peaked 14 d after MIA injection and resolved naturally after 28 d [36,37]. Therefore, we gave MIA stimulation to construct the KOA model on the 1st day and another MIA restimulation on the 28th day (Fig. 3A). Fourteen days after modeling, the contents of Il-1β, Il-6, and Tnf-α in serum were significantly increased compared with the first day, and large numbers of inflammatory cells infiltrated the synovial tissue, which confirmed the successful establishment of the KOA model (Fig. 3B and C). On the 28th day, the serum levels of the aforementioned proinflammatory factors returned to the levels observed on the 1st day, and the inflammatory cells in the synovial tissue had dissipated (Fig. 3B and C). The subsequent MIA restimulation up-regulated the serum levels of Il-1β, Il-6, and Tnf-α on the 42th day compared with the 14th day, indicating a stronger inflammatory response to the restimulation (Fig. 3B and C). Interestingly, although there was no statistical significance, serum level of the above proinflammatory factors were higher on the 56th day than on the 28th day, which may also indicate the progressive worsening of synovial inflammation in KOA with each flare, potentially promoting the progression of the disease (Fig. 3B and C). We also isolated circulating monocytes from the peripheral blood of mice and measured the relative protein expression levels of Il-1β, Il-6, and Tnf-α. The differences observed at various time points were entirely consistent with the changes in serum concentrations (Fig. 3D and E). Next, in order to study the expression differences during restimulation at the transcriptional level, we performed RNA-seq on circulating monocytes extracted at different time points. In principal component analysis (PCA) (Fig. 3F), there was partial overlap between the 1st-day cluster and the 28th-day cluster, indicating the inflammatory response subsided, and other clusters could be clearly distinguished. The heatmap shows the major differential genes at different time points, in which the gene differences between the 1st-day group and the 28th-day group were not significant, while the 14th-day group showed significant up-regulation of interleukin family, chemokine family, and alertin family proteins (Fig. 3G); the 42nd day showed further up-regulation of the expression levels of the aforementioned genes compared to the 14th day (Fig. 3H). KEGG analysis of DEGs between the 1st and 14th days (Fig. 3I), as well as between the 14th and 42nd days (Fig. 3J), revealed distinct transcriptional profiles associated with the onset of KOA and its subsequent flares in mice. The onset of KOA appeared to be more strongly enriched in inflammatory pathways (including the IL-17 signaling pathway and the TNF signaling pathway), whereas the transcriptional differences between the 2 flares also involved metabolism-related pathways, such as “Fatty acid metabolism” and “PPAR signaling pathway”.

Since circulating monocytes migrated to synovial inflammatory sites and participate in KOA under the action of chemokines, we designed an enzyme-linked immunosorbent assay (ELISA) array containing 21 common KOA chemokines, inflammatory cytokines, and alarm hormones (Fig. S3A). Serum levels of proinflammatory cytokines IL-1β, IL-6 IL-18, and TNF-α, chemokines CCL2, CCL3, and CCL4, alarm hormone HMGB1, S100A8, and S100A9 in KOA patients were significantly up-regulated compared with HD. Furthermore, we isolated human circulating monocytes and, after a 2-d resting period, restimulated them with IL-18, CCL4, or HMGB1. To our surprise, only HMGB1 induced a stronger inflammatory response in circulating monocytes derived from KOA patients, whereas stimulation with IL-18 or CCL4 did not produce any significant difference in the inflammatory response between monocytes from HD and those from KOA patients (Fig. S3B to D). Multiple large-scale clinical studies and experimental models have consistently identified HMGB1 as a key mediator in the pathogenesis of osteoarthritis (OA) [38,39]. Specifically, the concentration of HMGB1 in synovial fluid has been shown to correlate with radiographic severity scores, pain intensity ratings, and functional disability indices in patients with OA [40]. In our preliminary research, HMGB1 was found to be a critical risk factor for the onset and persistence of synovial inflammation. Our findings indicate that elevated HMGB1 levels are significantly associated with increased inflammatory cell infiltration and synovial hyperplasia in the affected joints, and inhibiting the expression of HMGB1 would help improve the state of synovial inflammation [11,41,42]. In summary, our results suggest that circulating monocytes derived from KOA patients acquire a trained phenotype and appear to exhibit immune memory specifically in response to HMGB1.

### HMGB1 serves as a specific DAMP that stimulates circulating monocytes in KOA mice to adopt the g7 phenotype and exhibit features of trained immunity

**Fig. 4.**
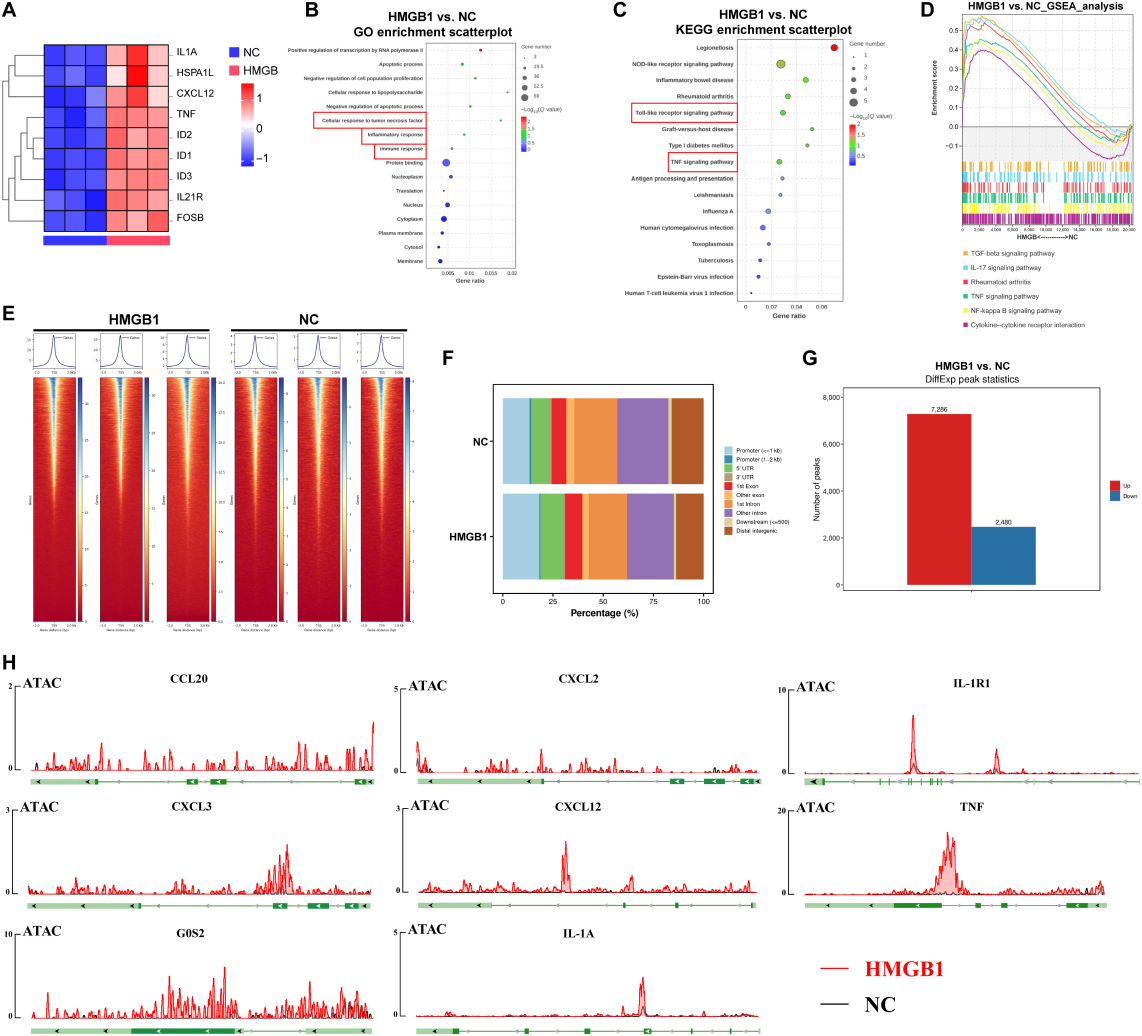
High mobility group protein B1 (HMGB1) serves as a specific damage-associated molecular pattern (DAMP) that stimulates circulating monocytes in knee osteoarthritis (KOA) mice to adopt the g7 phenotype and exhibit features of trained immunity. (A) Heatmap showing differences in the expression levels of inflammatory factors, chemokines, and alarmin family factors in circulating monocytes between the negative control (NC) group and the HMGB1 group in mice. (B) Gene Ontology (GO) enrichment analysis of differentially expressed genes in circulating monocytes between the NC and HMGB1 groups. (C) Kyoto Encyclopedia of Genes and Genomes (KEGG) enrichment analysis of differentially expressed genes in circulating monocytes between the NC and HMGB1 groups. (D) Gene set enrichment analysis (GSEA) of differentially expressed genes in circulating monocytes between the NC and HMGB1 groups. (E) The Cumulus heatmap shows the chromatin accessibility of circulating monocytes in the NC group and HMGB1 group mice. (F) Chromatin region occupancy in circulating monocytes of NC and HMGB1 group mice. (G) Bar graph statistics displaying the number of chromatin regions with differential accessibility (open or closed) in circulating monocytes from NC and HMGB1 group mice. (H) Chromatin accessibility of *IL1A*, *IL1R1*, *TNF*, *CXCL12*, *CXCL3*, *CXCL2*, *CCL20*, and *G0S2* genes in circulating monocytes of NC and HMGB1 group mice.

In the above study, HMGB1 can induce more release of proinflammatory factors from circulating monocytes of KOA patients. Take one step further, we stimulated monocytes with HMGB1 and conducted RNA-seq. Monocytes with or without HMGB1 stimulation can be clearly separated on the PCA plot (Fig. S4A). The heatmap illustrated the DEGs following HMGB1 treatment, among which *IL1A*, *CXCL12*, and *TNF* were significantly up-regulated, similar to the g7 population (Fig. 4A). We also found significant mRNA up-regulation of *IL1R1*, *CXCL3*, *CXCL2*, *CCL20*, and *G0S2* in HMGB1-induced monocytes (Fig. S4B). Gene Ontology (GO) enrichment analysis (Fig. 4B) indicated that differentially expressed genes were enriched in “inflammatory response”, “immune response”, and “cellular response to tumor necrosis factor”; in addition, KEGG analysis (Fig. 4C) indicated that DEGs enriched in “TNF signaling pathway” and “toll-like receptor signaling pathway”. In terms of gene set enrichment analysis (GSEA), large numbers of genes related to “NF-kappa B signaling pathway”, “TNF signaling pathway”, and “cytokine–cytokine receptor interaction” were activated in HMGB1-induced group (Fig. 4D). Furthermore, to better investigate whether HMGB1 stimulation can induce features of trained immunity—specifically chromatin accessibility—in monocytes, we performed assay for transposase-accessible chromatin with sequencing (ATAC-seq). We compared the correlation between negative control (NC) and HMGB1 and found a significant difference between the two (Fig. S4C). Venn diagram also illustrated large numbers of genes that differed between the two (Fig. S4D). The promoter region was further up-regulated, and the transcriptional state was further activated after HMGB1 stimulation (Fig. 4E), as well as an increase in chromatin-open genes (Fig. 4F and G), In particular, *IL1A*, *IL1R1*, *TNF*, *CXCL12*, *CXCL3*, *CXCL2*, *CCL20*, and *G0S2* exhibited greater chromatin accessibility upon HMGB1 stimulation (Fig. 4H). GO enrichment analysis (Fig. S4E) was mainly enriched in the “cellular component organization”, “cellular component organization or biogenesis”, and “regulation of signaling”, while KEGG enrichment analysis (Fig. S4F) was mainly enriched in the “Phosphatidylinositol signaling system”, “Focal adhesion”, and “Phospholipase D signaling pathway”. Overall, in this part, we found that HMGB1 serves as a specific DAMP that stimulates circulating monocytes in KOA mice to adopt the g7 phenotype and exhibit features of trained immunity.

### Trained circulating monocytes from KOA patients showed enhanced chromatin accessibility for the transcription factors IRF1 and IRF3

**Fig. 5.**
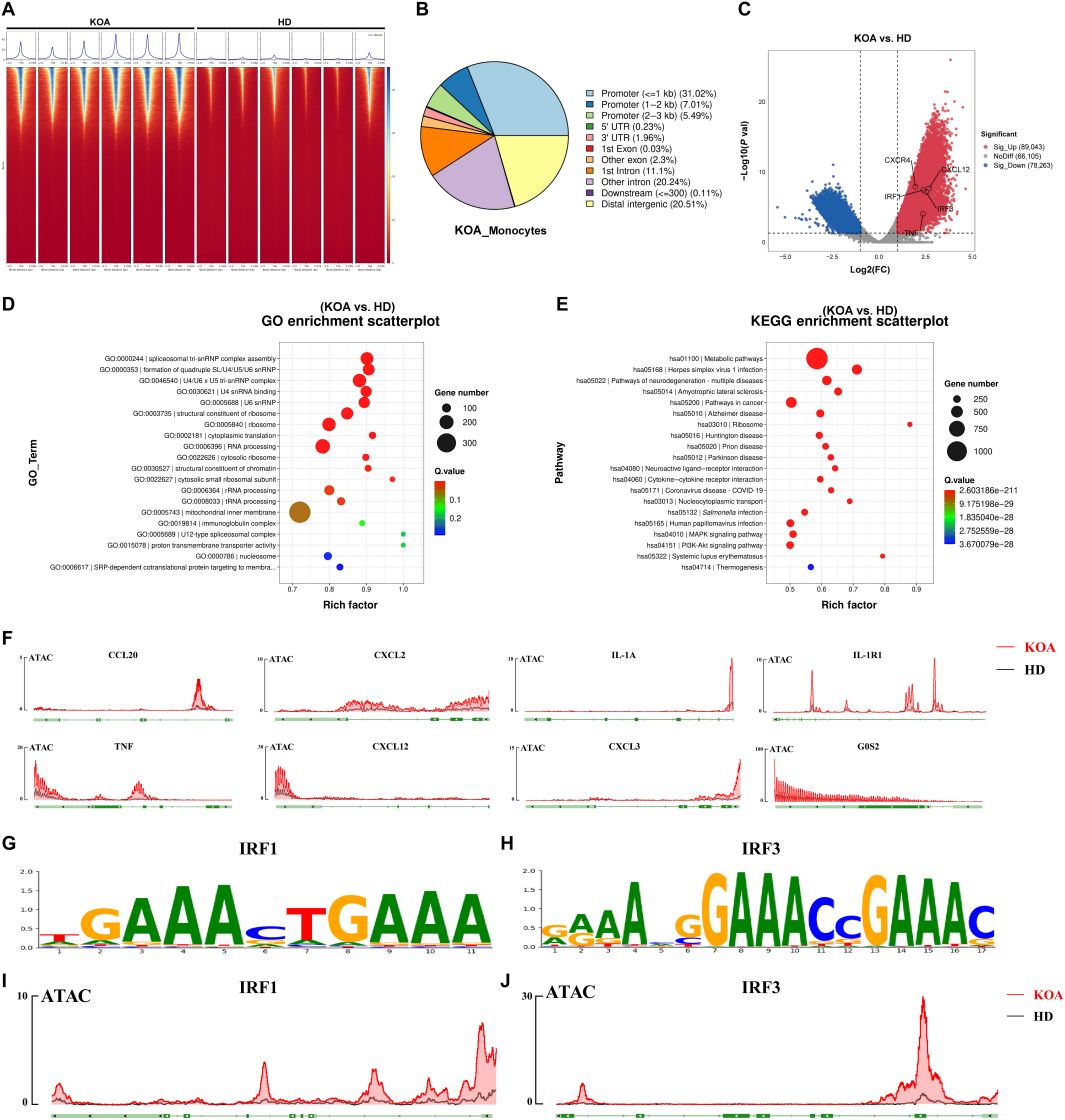
Trained circulating monocytes from knee osteoarthritis (KOA) patients showed enhanced chromatin accessibility for the transcription factors interferon regulatory factor 1 (IRF1) and IRF3. (A) Cumulus heatmaps display the chromatin accessibility in peripheral monocytes of the healthy donor (HD) group and KOA group. (B) Percentage of chromatin regions in peripheral monocytes of the KOA group. (C) Volcano plot illustrating the differences in gene chromatin accessibility between peripheral monocytes of the HD and KOA groups. (D) Gene Ontology (GO) enrichment analysis of gene chromatin accessibility differences in peripheral monocytes between the HD and KOA groups. (E) Kyoto Encyclopedia of Genes and Genomes (KEGG) enrichment analysis of gene chromatin accessibility differences between the HD and KOA groups in peripheral monocytes. (F) Differences in chromatin accessibility of the *IL1A*, *IL1R1*, *TNF*, *CXCL12*, *CXCL3*, *CXCL2*, *CCL20*, and *G0S2* genes in peripheral circulating monocytes between the HD and KOA groups. (G) Motif analysis of the IRF1 gene in peripheral monocytes between the HD and KOA groups. (H) Motif analysis of the IRF3 gene in peripheral monocytes between the HD and KOA groups. (I) Comparison of chromatin accessibility intensity of IRF1 in peripheral monocytes between the HD and KOA groups. (J) Comparison of chromatin accessibility intensity of IRF3 in peripheral monocytes between the HD and KOA groups.

Given the findings regarding HMGB1-induced g7-trained phenotype in mouse monocytes, we revisited the single-cell sequencing datasets from both HD and KOA patients. Through the GSEA for g7 in monocytes (Fig. S5A), we found that the differences between the 2 groups were mainly concentrated in the “chromatin organization”, “mRNA processing”, “nuclear speck”, and “RNA slicing”. Notably, earlier studies have indicated that HMGB1 may interact with CXCL12 to form a complex that facilitates the chemotactic migration of CXCR4^+^ cells [43]. Based on the above evidence, we isolated CXCR4^+^ circulating monocytes from HD and KOA patients and performed ATAC-seq analysis. At the global level, the distribution of called peaks in KOA patients is concentrated in close proximity to the transcription start site, completely different from healthy people (Fig. 5A). To further explore the differences between the 2 groups of samples at the level of chromatin openness, we used PCA for downscaling to observe the differences in their components, and the results showed that the samples between the 2 groups differed significantly in the PCA (Fig. S5B). Heatmaps were plotted using dimensionality-decreasing cluster analysis to show the difference in chromatin opening levels between the HD and KOA groups. We can find that the chromatin structure of genes within the KOA group is more open than that of the HD group (Fig. S5C). By mapping ATAC-seq peaks to genomic regions, we found that ~43% of peaks mapped within the putative promoter and ~20% were in distal intergenic regions (Fig. 5B). The enrichment of accessible chromatin in intergenic regions suggests that distal regulatory sequences, such as enhancers, noncoding regulatory elements in cis, can be accessed to regulate gene expression. In many cases, peaks located mainly in promoters, but also, those harbored in the gene body (introns and exons) have been found to be good predictors of gene expression [44]. Therefore, our results indicated increased chromatin accessibility of CXCR4^+^ monocyte in KOA patients. By comparing ATAC-seq peaks, we found 167,666 differentially accessible chromatin regions; among these, 89,043 were identified in gene promoters in the KOA patients, such as *CXCR4*, *CXCL12*, *IRF1*, and *IRF3* (Fig. 5C). We observed a striking number of inflammation-related differentially accessible chromatin regions likely consistent with synovial inflammatory response in KOA. GO enrichment analysis (Fig. 5D) indicated that DEGs were enriched in “structural constituent of ribosome”, “structural constituent of chromatin”, and “nucleosome”. KEGG analysis (Fig. 5E) indicated that DEGs were enriched in “pathway of neurodegeneration”, “ribosome”, and “cytokine–cytokine receptor interaction”. Consistent with the g7-trained phenotype, *IL1A*, *IL1R1*, *TNF*, *CXCL12*, *CXCL3*, *CXCL2*, *CCL20*, and *G0S2* exhibited greater chromatin accessibility in CXCR4^+^ circulating monocytes from KOA patients (Fig. 5F).

Previous reports have shown that MyD88 acts as a direct downstream target of HMGB1, subsequently initiating transcriptional regulation through multiple transcription factors. IRF1, the eponymous member of the IRF family, was originally identified as a nuclear factor that binds and activates the promoters of type I IFN, ubiquitously expressed in human cells at low basal levels and highly responsive to a variety of stimuli, including MyD88 [45]. IRF1 mediates the transcription of a multitude of proinflammatory genes by binding to “GAAA” sites within cis-regulatory promoter and enhancer elements to contribute dynamic transcriptional activation networks. Our results demonstrated the active opening of “GAAA” sites in CXCR4^+^ monocyte of KOA patients. Similarly, often as a downstream signal of IRF1, IRF3 also exhibited binding activity at the “GAAA” site (Fig. 5G to I). Together, these findings demonstrate that trained circulating monocytes from KOA patients show increased chromatin accessibility for the transcription factors IRF1 and IRF3.

### HMGB1 promotes IRF1 SUMOylation via MyD88 to induce trained immunity in KOA monocytes

**Fig. 6.**
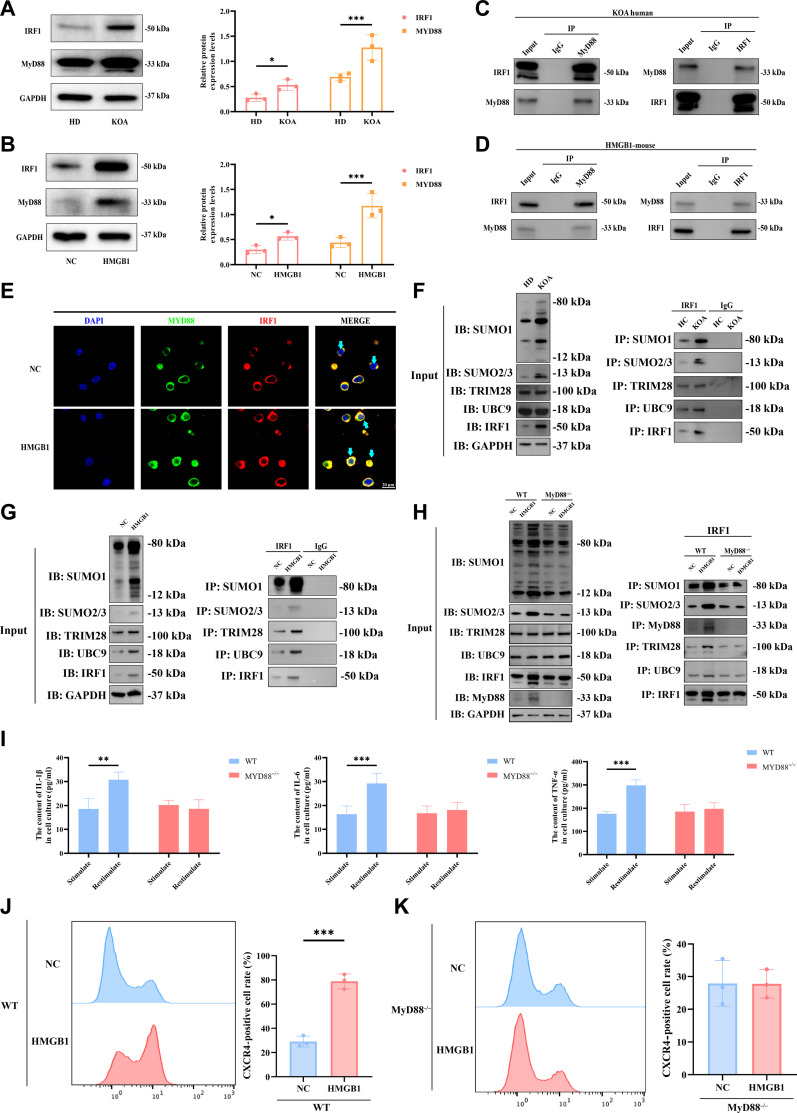
High mobility group protein B1 (HMGB1) promotes interferon regulatory factor 1 (IRF1) SUMOylation via MyD88 to induce trained immunity in knee osteoarthritis (KOA) monocytes. (A) Western blotting (WB) was used to detect the expression levels of IRF1 and MyD88 proteins in peripheral blood monocytes from patients in the healthy donor (HD) and KOA groups (*n* = 3). (B) WB was used to detect the expression levels of IRF1 and MyD88 proteins in peripheral blood monocytes from mice in the negative control (NC) and HMGB1 groups (*n* = 3). (C) Coimmunoprecipitation (CO-IP) was used to examine the interaction between IRF1 and MyD88 proteins in peripheral blood monocytes from patients in the HD and KOA groups. (D) CO-IP was used to examine the interaction between IRF1 and MyD88 proteins in peripheral blood monocytes from mice in the NC and HMGB1 groups. (E) A laser confocal microscope was used to observe the interaction between IRF1 and MyD88 proteins in peripheral blood monocytes from mice in the NC and HMGB1 groups. (F) CO-IP was used to evaluate the SUMOylation of IRF1 in peripheral blood monocytes from patients in the HD and KOA groups. (G) CO-IP was used to evaluate the SUMOylation of IRF1 in peripheral blood monocytes from mice in the NC and HMGB1 groups. (H) CO-IP was used to compare the differences in SUMOylation levels of IRF1 in peripheral blood monocytes from wild-type and MyD88 knockout mice after HMGB1 stimulation. (I) Enzyme-linked immunosorbent assay (ELISA) was used to measure the levels of interleukin-1β (IL-1β), IL-6, and tumor necrosis factor-α (TNF-α) in peripheral blood monocytes from wild-type and MyD88 knockout mice after HMGB1 stimulation (*n* = 6). (J) Flow cytometry was used to determine the proportion of CXCR4-positive cells in peripheral blood monocytes from wild-type mice stimulated with HMGB1 (*n* = 3). (K) Flow cytometry was used to determine the proportion of CXCR4-positive cells in peripheral blood monocytes from MyD88 knockout mice stimulated with HMGB1 (*n* = 3). Statistical results are represented by mean ± standard error of the mean (mean ± SEM), *P < 0.05, **P < 0.01, ***P < 0.001.

Up to this point, our findings indicated that HMGB1 mediates the trained immunity of circulating monocytes in KOA, leading to the emergence of a special g7 subgroup. This subgroup showed chromatin structure remodeling associated with inflammation-related transcription factors such as IRF1. However, how HMGB1 forms circulating monocytes-trained immunity was still unknown. MyD88 is a direct downstream signaling molecule when HMGB1 activates Toll-like receptors. The protein levels of MyD88 and IRF1 in circulating monocytes of KOA patients were significantly higher than those of HD (Fig. 6A), as well as in HMGB1-induced THP-1 compared with NC (Fig. 6B). Next, we confirmed the interaction between MyD88 and IRF1 (Fig. 6C and D). Immunoblotting results showed the presence of MyD88 in IRF1 immunoprecipitation, and conversely, the presence of IRF1 in MyD88 immunoprecipitation. Previous studies have shown that MyD88 can carry IRF1 from the cytoplasm and transfer to the nucleus [46]; then, we observed the stimulation of HMGB1 caused the colocalization of MyD88 with IRF and the transport to the nucleus with a laser confocal microscopy (Fig. 6E). Traditionally, due to the short half-life of IRF1 protein, the stability on the nucleus and their functions requires further protein structural modification, such as SUMOylation, a highly dynamic ubiquitin-like protein modification requiring the participation E1 class activating enzyme, E2 conjugating enzyme Ubc9, and SUMO E3 ligases [47,48]. We searched the BioGRID database and predicted that TRIM28 may be the SUMO E3 ligases of MyD88-IRF1. Subsequently, by comparing circulating monocytes from KOA patients and HD, as well as THP-1 stimulated with or without HMGB1, we identified both SUMO1 and SUMO2/3 involved in and showed enhanced SUMOylation levels under the KOA environment (Fig. 6F and G). Using SUMO, SUMO2/3, Ubc9, TRIM28, or IRF1 affinity beads respectively, we detected that the enhanced IRF1-conjugate levels under the KOA environment or stimulated with HMGB1 (Fig. 6F and G). On the other hand, circulating monocytes from MyD88^−/−^ mice lost the ability to enhance SUMOylation induced with HMGB1, while protein interactions including Ubc9, TRIM28, MyD88, and IRF1 were also weakened (Fig. 6H). Besides, when circulating monocytes from MyD88^−/−^ mice were restimulated with HMGB1, they released IL-1β, IL-6, and TNF-α at a significantly lower level than the initial stimulation (Fig. 6I), and their ability to differentiate into g7 or CXCR4^+^ subgroup under HMGB1 stimulation also disappeared (Fig. 6J and K and Fig. S6).

### Inhibition of HMGB1 eliminated the trained immunity of circulating monocytes and their aggregation to KOA synovial tissue

**Fig. 7.**
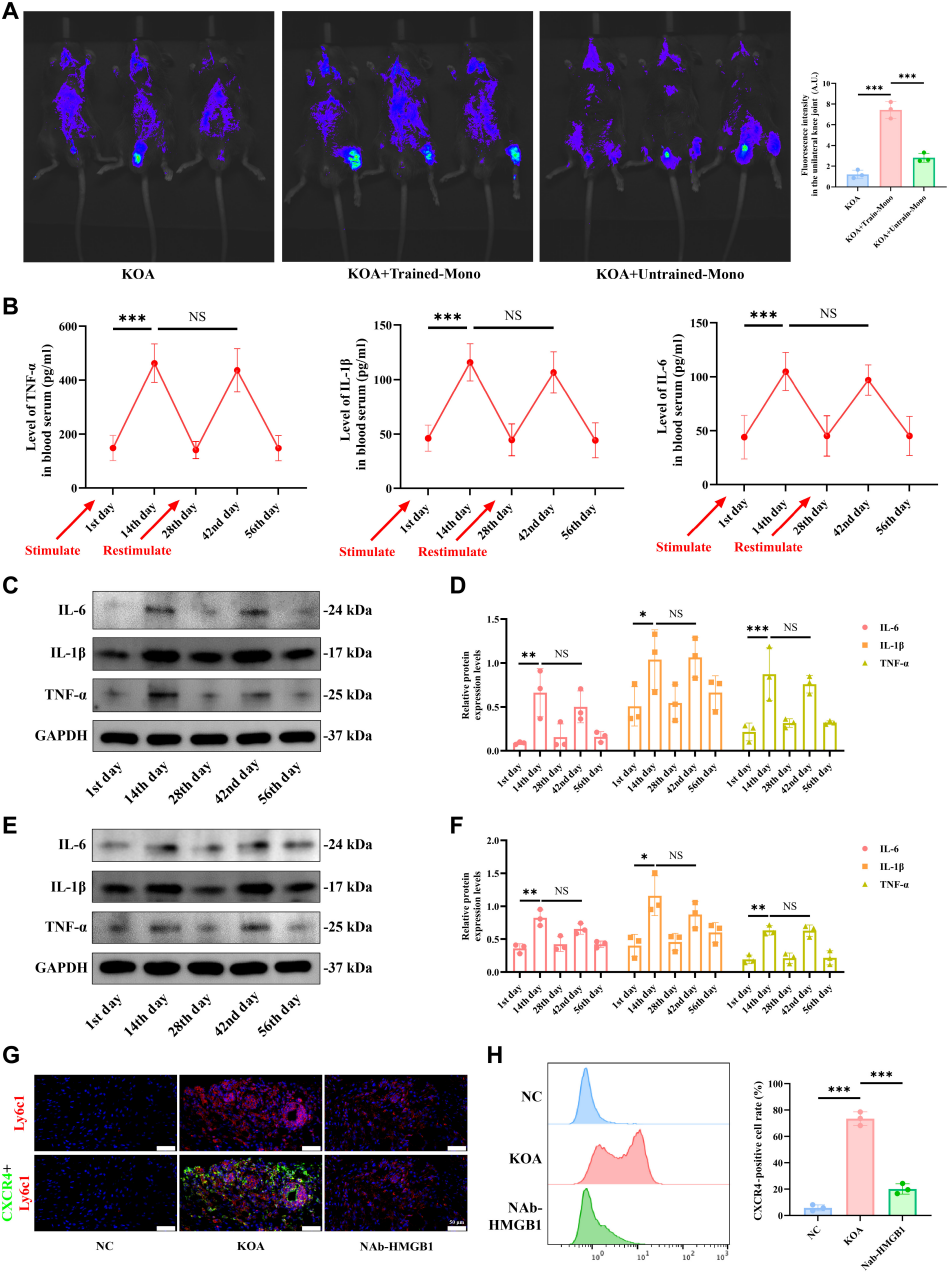
Inhibition of high mobility group protein B1 (HMGB1) eliminated the trained immunity of circulating monocytes and their aggregation to knee osteoarthritis (KOA) synovial tissue. (A) In vivo imaging was used to observe the locations of cell migration in the KOA group, the KOA group with transfused fluorescently labeled trained circulating monocytes, and the KOA group with transfused fluorescently labeled untrained circulating monocytes in mice (*n* = 3). (B) Enzyme-linked immunosorbent assay (ELISA) was performed to measure the levels of interleukin-1β (IL-1β), IL-6, and tumor necrosis factor-α (TNF-α) in mouse peripheral serum at different time points under Nab-HMGB1 intervention (*n* = 6). (C) Western blotting (WB) was used to detect the expression of IL-1β, IL-6, and TNF-α in mouse synovial tissue at different time points following Nab-HMGB1 intervention. (D) Representative WB bands showing the levels of IL-1β, IL-6, and TNF-α in peripheral circulating monocytes from mice at different time points under Nab-HMGB1 intervention (*n* = 3). (E) Statistical graphs of the WB results for IL-1β, IL-6, and TNF-α levels in peripheral circulating monocytes from mice at different time points under Nab-HMGB1 intervention. (F) Representative WB bands showing the levels of IL-1β, IL-6, and TNF-α in peripheral circulating monocytes from mice at different time points under Nab-HMGB1 intervention (*n* = 3). (G) Immunofluorescence was used to observe the numbers of CXCR4^+^ and Ly6c^+^ cells in synovial tissue from the negative control (NC) group, KOA group, and Nab-HMGB1 group of mice. (H) The number of CXCR4^+^ circulating monocytes in mouse synovial tissue from the NC group, KOA group, and Nab-HMGB1 group was detected using magnetic bead sorting of mouse circulating monocytes followed by flow cytometry (*n* = 3). Statistical results are represented by mean ± standard error of the mean (mean ± SEM), *P < 0.05, **P < 0.01, ***P < 0.001. NS, not significant.

After clarifying the mechanistic pathway by which HMGB1 induces trained immunity in circulating monocytes, we began to investigate whether these cells are involved in synovial inflammation in KOA. First, we labeled trained and untrained circulating monocytes from mice and transfused them into a KOA model. In vivo imaging data demonstrated that trained, but not untrained, monocytes accumulated in the KOA knee joint (Fig. 7A). Besides, on the 42th day, although MIA restimulation still caused increased serum levels of IL-1β, IL-6, and TNF-α in KOA mice treated with HMGB1 neutralization antibody, it was no longer statistically significant compared to the 28th day (Fig. 7B). The relative expression levels of IL-1β, IL-6, and TNF-α in circulating monocytes (Fig. 7C and D) and synovial tissues (Fig. 7E and F) at the 2 aforementioned time points were consistent with the trends observed in their serum concentrations. In addition, on the 14th day, the fluorescence intensity of CXCR4^+^ monocytes in the synovial tissue of KOA mice were significantly increased compared to the NC group, whereas administration of an HMGB1-neutralizing antibody inhibited this cellular accumulation (Fig. 7G). Consistently, flow cytometry analysis showed that administration of an HMGB1-neutralizing antibody significantly reduced the percentage of CXCR4^+^ monocytes in dissociated synovial tissues from KOA mice (Fig. 7H). Meanwhile, the results of hematoxylin and eosin (HE) and Safranin O staining of knee joint cartilage tissues from various groups of mice indicate that the inhibition of HMGB1 significantly reduced matrix damage and pathological progression in cartilage tissue (Fig. S7A).

### CXCR4-dependent migration of trained subsets promoted synovial inflammation in KOA

**Fig. 8.**
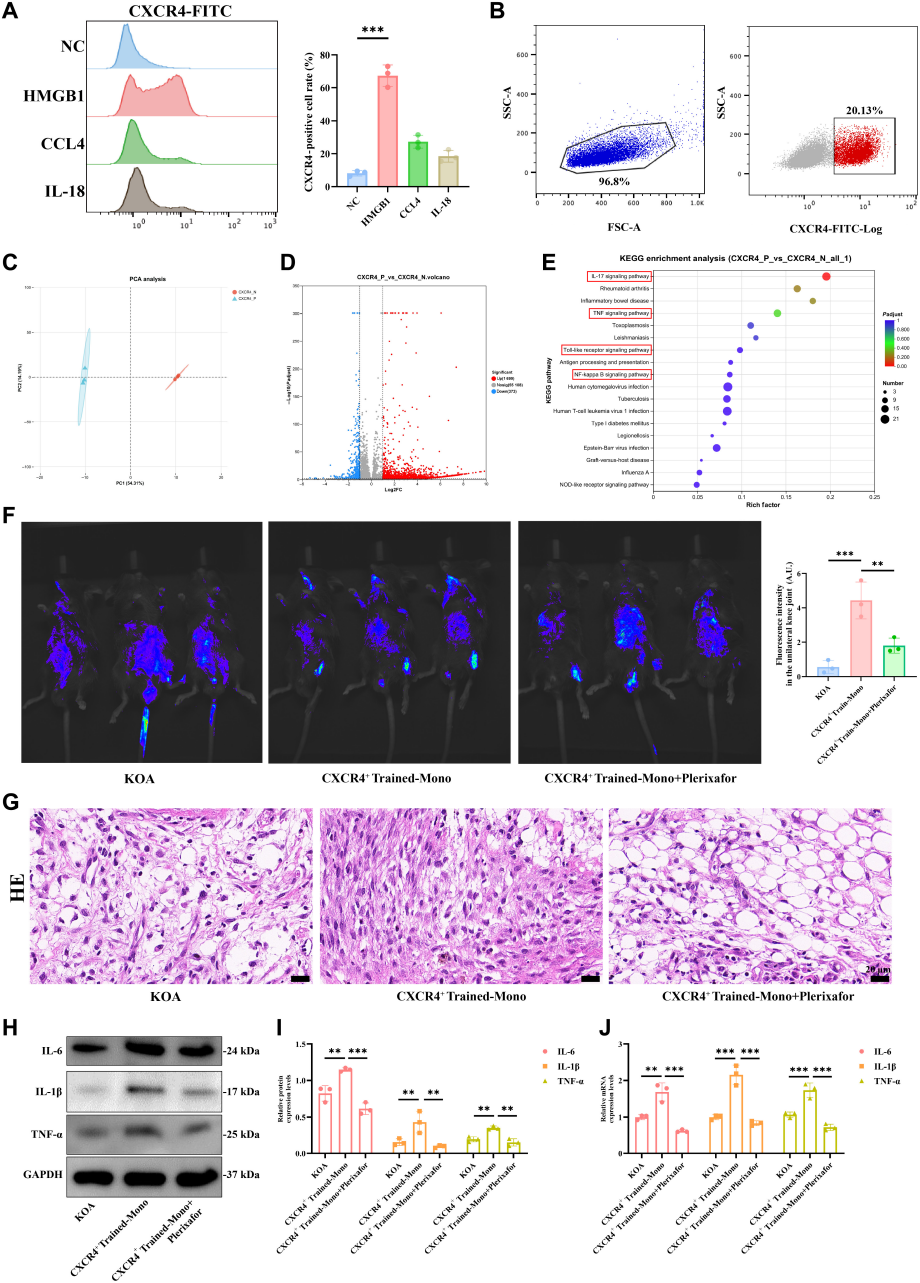
CXCR4-dependent migration of trained subsets promoted synovial inflammation in knee osteoarthritis (KOA). (A) Flow cytometry was used to assess the effects of high mobility group protein B1 (HMGB1), CCL4, and interleukin-18 (IL-18) treatments on the proportion of CXCR4-positive cells among circulating monocytes in mice (*n* = 3). (B) CXCR4^+^ cells were isolated using flow cytometric sorting. (C) Principal component analysis (PCA) was performed on CXCR4^+^ and CXCR4^−^ cells. (D) A volcano plot displays the number of differentially expressed genes between CXCR4^+^ and CXCR4^−^ cells. (E) Kyoto Encyclopedia of Genes and Genomes (KEGG) database enrichment analysis was conducted for the differentially expressed genes between CXCR4^+^ and CXCR4^−^ cells. (F) In vivo animal fluorescence imaging demonstrates the effects of transfusing CXCR4^+^ cells and administering the CXCR4 receptor antagonist plerixafor on CXCR4^+^ cells in KOA mice (*n* = 3). (G) Hematoxylin and eosin (HE) staining was used to observe synovial pathology in the KOA group, the CXCR4^+^ cell transfusion group, and the CXCR4^+^ cell transfusion plus plerixafor group. (H) Representative Western blot images show protein expression levels of IL-1β, IL-6, and tumor necrosis factor-α (TNF-α) in the synovial tissue of mice from the KOA group, CXCR4^+^ cell transfusion group, and CXCR4^+^ cell transfusion plus plerixafor group. (I) Quantitative Western blot analysis of IL-1β, IL-6, and TNF-α protein expression levels in synovial tissue from the 3 groups (*n* = 3). (J) Quantitative polymerase chain reaction (PCR) analysis of IL-1β, IL-6, and TNF-α mRNA expression levels in synovial tissue from the KOA group, CXCR4^+^ cell transfusion group, and CXCR4^+^ cell transfusion plus plerixafor group (*n* = 3). Statistical results are represented by mean ± standard error of the mean (mean ± SEM), **P < 0.01, ***P < 0.001.

Finally, we investigated whether CXCR4 is specifically involved in the recruitment of the g7-trained monocyte subpopulation to synovial tissue and its impact on synovial inflammation in KOA. We first examined the proportion of CXCR4-positive cells among HMGB1-trained monocytes by flow cytometry and subsequently isolated this population of CXCR4^+^-trained monocytes (Fig. 8A and B). PCA plot of the RNA-seq data showed a distinct clustering between CXCR4^+^- and CXCR4^−^-trained monocytes (Fig. 8C). A total of 2,072 DEGs were identified between these 2 groups, with 1,696 genes significantly up-regulated (Fig. 8D). Hierarchical clustering of the identified DEGs revealed distinct collections of genes, with subcluster 2 being specifically associated with the proinflammatory response and primarily enriched in the “Cytokine–cytokine receptor interaction” pathway (Fig. S8A to C). We also took the terms analyzed by GO and KEGG in g7 as the reference map and confirmed that DEGs between CXCR4^+^- and CXCR4^−^-trained monocytes were significantly enriched in most of the above terms, especially “regulation of response to stimulus” in GO analysis (Fig. S8D) and “NF-kappa B signaling pathway”, “TNF signaling pathway”, and “toll-like receptor signaling pathway” in KEGG (Fig. 8E). In terms of GSEA, large numbers of genes related to “TNF-α signaling via NF-κB” were activated in CXCR4^+^-trained monocytes (Fig. S8E). Together, the RNA-seq findings indicate that CXCR4^+^-trained monocytes exhibit functional similarities to the subgroup g7 circulating monocytes identified in KOA patients. Following this, we injected CXCR4^+^-trained monocytes into KOA mice, these cells specifically migrated to the KOA synovium, and their migration was inhibited by plerixafor, a specific CXCR4 blocker (Fig. 8F). In addition, CXCR4^+^-trained monocytes induced more severe synovial inflammation and significantly increased the protein expression levels of proinflammatory factors, including Il-1β, Il-6, and Tnf-α. Notably, treatment with plerixafor effectively blocked the proinflammatory effects of CXCR4^+^-trained monocytes on KOA synovial tissue (Fig. 8G to J).

## Discussion

Several decades of synovial tissue research together with recent technical advances now allow an in-depth look and characterization of monocytes subsets within the healthy and KOA inflamed synovial tissue. Circulating monocytes constantly enter the tissues and maintain the pool of local macrophages during both steady state and inflammation. On this occasion, monocytes or macrophages represent heterogeneous populations and display a substantial degree of plasticity [49–51]. For instance, M1 macrophages represent a proinflammatory type characterized by the production of proinflammatory cytokines and matrix metalloproteinase, while M2 macrophages show an elevated expression of scavenger receptors such as CD163 and surface receptors such as CD206 and reduced production of proinflammatory cytokines [52]. Notably, both of these cell subtypes are monocyte-derived and are extreme somehow. Monocytes should have undergone a greater variety of fate choices during recruitment and migration by the inflammatory synovial membrane. In this context, inflammatory CCR2^+^ Ly6C^+^ in mice or CD14^+^ CD16^−^ in human is the most basic classification of monocytes in KOA. Their infiltration into KOA synovium is associated to clinical disease parameters and skew a proinflammatory phenotype shift in residential synovial macrophages, thus pushing the overall synovial inflammation toward disease progress [53]. Recent researches supported the idea that monocytes or monocyte-derived macrophages are important drivers of KOA-progressive synovial inflammation. Higher expression of the chemokines CCL2, CCL3, and CCL4 in KOA synovium recruits circulating monocytes via the CCL2/CCR2 or CCL3/ CCR1 axis [28,54]. In this study, we identified a special subgroup (g7) of circulating monocytes from KOA patients, highly expressing *IL1A*, *IL1R1*, *TNF*, *CXCL12*, *CXCL3*, *CXCL2*, *CCL20*, and *G0S2*. This cluster had epigenetic transcriptional reserves of proinflammatory factors and would release more IL-1β, IL-6, and TNF-α when challenged with restimulation, thus promoting the progression of KOA synovial inflammation.

The inflammatory sensitivity of g7 is highly consistent with the trained phenotype described as previous. This phenomenon called “innate immune memory” or “trained immunity” transformed our understanding of chronic inflammation and autoimmunity. Growing evidence clearly indicated how the altered cytokine milieu in inflammatory disorders might promote a “priming” or “training” of macrophages and implicate the existence of residual innate transcriptional memory [55,56]. As far as KOA synovial inflammation is concerned, “danger signals” including HMGB1, S100 proteins, fragmented hyaluronic acid, or heat shock proteins are abundantly present within the synovial fluid and the joint cavity and potentially represent triggers of inflammation that are continuously present [57]. These DAMPs often induce immune response through Toll-like receptor or nod-like receptor, these DAMPs usually induce immune response through Toll-like receptors or nucleotide-binding oligomerization domain-like receptors, resulting in large numbers of proinflammatory factor synthesis. However, in the context of trained immunity, DAMP stimulation not only has an impact on the present but also induces the transcriptional reserve of proinflammatory factors, makes the body sensitive to inflammation, and exhibits a more intense inflammatory response in the face of the next stimulus [58]. Systematic analysis of clinical serum samples from KOA patients at different disease stages has yielded compelling evidence, revealing a significant pattern: HMGB1 exhibits greater concentration fluctuations and a wider dynamic range compared to S100A8/A9. Specifically, longitudinal measurements indicate that HMGB1 serum levels can differ by several fold between early- and late-stage KOA patients, whereas other DAMPs like S100A8/A9 typically show a more modest and relatively stable elevation during disease progression [39]. Furthermore, compared to other simultaneously measured DAMPs, changes in HMGB1 concentration show a stronger correlation with acute inflammatory episodes, pain exacerbation events, and markers of radiographic progression [40,59]. In this study, we observed the training effect of HMGB1 on circulating monocytes. HMGB1 induced the differentiation of monocytes into g7 subgroup and gained the trained phenotype.

HMGB1, a ubiquitous nuclear protein conserved across evolution from archaea to mammals, is a necessary and sufficient mediator of sterile injury and infection-elicited inflammation and immunity [60]. By activating the receptor for advanced glycation end products or Toll-like receptors 2, 4, and 9, HMGB1 initiates intracellular signaling, resulting in increased cytokine expression and inflammation. HMGB1 expression levels were elevated in serum, synovial tissue, or synovial fluid in KOA patients compared to healthy subjects [59,61]. Besides, administration of HMGB1 antagonists significantly attenuates the severity of inflammation in inflammatory bowel disease, arthritis, and neuroinflammation [62]. In our study, HMGB1 induced a trained phenotype in monocytes and enhanced their expression of CXCR4, which subsequently led to their specific migration to synovial tissue and progressively aggravated synovial inflammation. Consistently, previous reports showed an unexpected CXCR4 retention on the cell surface triggered by the CXCL12/HMGB1 heterocomplex, promoting the availability of CXCR4 and enhancing the directional cell migration.

Mechanistically, the induction of trained immunity has been closely associated with an active interplay between epigenetic and metabolic reprogramming. Indeed, macrophages exposed to proinflammatory stimuli are characterized by an enhanced glycolytic influx and a fragmented tricarboxylic acid cycle, leading to the accumulation of intermediate metabolites such as succinate and acetyl coenzyme A that serve as cofactors for epigenetic remodeling enzymes. The analysis of circulating monocytes and synovial macrophages in patients with inflammatory arthritis has demonstrated increased glycolysis and an altered fatty acid metabolism, which could explain the accumulation of intermediate metabolites such as succinate and glutamate in the synovial fluid of inflamed joints [63]. Importantly, these metabolic abnormalities correlate with an enhanced capacity of arthritic monocytes to release proinflammatory factors upon ex vivo stimulation. Besides, this provides another theoretical explanation for the involvement of monocyte-trained immunity in the progression of synovial inflammation in KOA. In our study, however, we identified the chromatin domain open in IRF1 via ATAC-seq. Traditionally, IRF1 is preferably induced by type I IFNs instead of type III IFNs (IFN-III) and decides the proinflammatory response mediated by IFN-I, but Toll-like receptors can also activate IRF1 via downstream MyD88, as well as TNF receptor signaling [64]. In recent years, studies have consistently revealed that transcriptional activation of IRF1 promotes cartilage injury in KOA [65]. Notably, IRF1 can also act cooperatively with other transcription factors to induce gene expression. Distinct sets of genes have been shown to be transcriptionally controlled by IRF1 acting alone versus when it is complexed with IRF8. It is likely that the transcriptional spectrum is similarly influenced, with different results, when bound to DNA as a heterodimer complexed with other transcription factors, such as IRF2, nuclear factor-κB, or signal transducer and activator of transcription 1. Hence, the binding of IRF1 to individual IRF-E and interferon-stimulated response element sites in concert with other available transcription factors may dynamically shape the transcriptional status of the genome [66]. These dynamic changes are likely to be particularly prominent as additional transcription factors become activated during the transition from basal, constitutive IRF1-mediated transcription of host defense genes to higher-level responses induced by pattern recognition receptor-initiated signaling. Dimer-specific binding sites exist for IRF3, IRF5, or IRF7 homodimers, and it seems likely that they exist for IRF1 homodimers as well. Meanwhile, through systematic bioinformatics analysis and data mining of existing public databases, we have discovered a large body of high-quality transcriptomic and epigenetic evidence. This evidence consistently reveals an important biological phenomenon: Peripheral blood circulating monocytes treated with trained immunity exhibit gene expression profiles and regulatory network patterns highly similar to our research findings [67]. It is particularly noteworthy that within these differentially expressed gene networks, IRF1 acts as a core transcriptional regulatory factor, playing a crucial and dominant role [68]. IRF1 may function as a key “molecular switch” in the trained immunity response, coordinating and integrating multiple signaling pathways to drive the transformation of monocytes toward a proinflammatory phenotype.

Epigenetic modifications dynamically regulate the state of chromatin, thereby influencing the binding of IRF1 and its cofactors to promoter elements, and this could be the potential mechanism for memory traits of trained immunity [69]. In our study, activated MyD88 interacts with IRF1 in the cytoplasm and begins to migrate to the nucleus and subsequently stabilizes in the nucleus through SUMOylation. SUMOylation is a common ubiquitin-like protein modification, and the process of linking SUMO molecules to lysine residues of target proteins, which is a covalent binding involving E1 activating enzyme, E2 binding enzyme, and E3 ligase cascade, and ultimately changes the structure and function of the substrate target protein, affecting its localization, stability, and activity in cells. We searched the BioGRID database and predicted that TRIM28 may be the SUMO E3 ligases of MyD88-IRF1. Subsequently, by comparing circulating monocytes from KOA patients and HD, as well as THP-1 stimulated with or without HMGB1, we identified both SUMO1 and SUMO2/3 involved in and showed an enhanced SUMOylation levels under the KOA environment.

However, this study still has several limitations. We acknowledge that the sample size of this research is relatively small, with only 6 participants in each group. This limitation may lead to insufficient statistical power, unstable effect size estimates, and wider confidence intervals, thereby increasing the risk of false negatives and false positives. In future clinical research, we will expand the sample size, prioritizing a multicenter, prospective design, and emphasize effect sizes and their confidence intervals in our reporting to enhance the reliability and generalizability of the results. Additionally, future research will further include experiments on the “functional deficiency” of MyD88 to substantiate its crucial role in trained immunity. Although the MIA model is widely used in the study of KOA pain and structural damage due to its advantages such as simple operation, short modeling cycle, and high reproducibility of experimental results, there are still significant differences between its pathophysiology and that of human chronic KOA. In our subsequent work, we will combine surgically induced models, spontaneous OA models, metabolic OA models, and large-animal models to verify pathological relevance and clinical translation. We will also use multiomics technologies to establish a cross-species comparative database to clarify the scope of application and limitations of each model. This will improve the success rate of translating basic research into clinical applications and avoid overinterpretation resulting from using the MIA model as the sole basis for clinical translation. Furthermore, factors such as gender and age have been shown to influence the function and phenotype of immune cells. Therefore, in this study, to further mitigate the impact of these factors when investigating immune phenotypes, we will apply baseline normalization during the collection of clinical samples and the establishment of animal models.

In summary, we combined single-cell sequencing, ATAC-seq, and RNA-seq analyses to identify a specific subpopulation from circulating monocytes of KOA patients. They exhibited high expression levels of IL1A, IL1R1, TNF, CXCL12, CXCL3, CXCL2, CCL20, and G0S2, demonstrated a memory response to previous stimuli, specifically migrated to synovial tissue, and released elevated levels of inflammatory factors upon subsequent stimulation. In addition, we find that MyD88, downstream of HMGB1, recruits cytoplasmic IRF1 to the nucleus and then stabilizes IRF1 in chromatin through SUMOylation of TRIM28 to exert transcriptional activity and epigenetic enhancement of proinflammatory factor expression. Furthermore, CXCR4 plays a central role in directing the migration of trained phenotype monocytes toward synovial tissue in KOA. Taken together, this study reveals that HMGB1 promotes the SUMOylation of IRF1 via MyD88 signaling to activate trained immunity in circulating monocytes, thereby exacerbating synovial inflammation in KOA.

## Methods

### Laboratory animals and modeling

Wild-type C57BL/6J male mice (6 to 8 weeks old) were purchased from Jiangsu Huanoxinchuang Co, Ltd., and MyD88 knockout C57BL/6J mice (6 to 8 weeks old) were purchased from Jiangsu Jicui Yaokang Biotechnology Co, Ltd. The animals were maintained in a specific-pathogen-free animal room with a temperature of 26 ± 0.5 °C, humidity of 50% ± 5%, and a 12-h light-dark cycle, with free access to water and food. We utilized a microinjection syringe (Hamilton) to inject 1 mg of iodacetic acid (I104613, Shanghai Aladdin Biochemical Technology Co., Ltd.) into the unilateral knee joint to establish a unilateral KOA model, with stimulation conducted on day 0 and day 28, respectively. This animal experiment was approved by the Animal Ethics Committee of Nanjing University of Traditional Chinese Medicine (approval number: 2024NL-KS095).

### Clinical sample collection

The peripheral blood mononuclear cell (PBMC) study was approved by the Medical Ethics Committee of the Jiangsu Provincial Hospital of Traditional Chinese Medicine (approval number: 2022NL-180-01). Patients with KOA (*n* = 6, male) and healthy volunteers (HD) (*n* = 6, male) were recruited from the Jiangsu Provincial Hospital of Traditional Chinese Medicine, and PBMCs were obtained by peripheral blood collection. Exclusion criteria included a history of diabetes, arterial hypertension, chronic renal failure, signs or history of infection at the time of inclusion, or any history or clinical symptoms of inflammatory disease. It was also ensured that the HD had no history of back pain, arthritis, joint damage, or other inflammation. Written informed consent was obtained from all participants. All samples were collected in a fasting state at 9:00 AM to ensure that the samples were sourced at the same time point and condition. Detailed baseline characteristics are presented in Table 1.

**Table 1.** Comparison of baseline data from 2 groups of clinical samples

Group	*n*	Age/years	BMI/(kg·m^−2^)	Kellgren–Lawrence grading system
HD	6	57.50 ± 1.04	25.93 ± 0.31	0
KOA	6	57.67 ± 1.63	25.35 ± 1.00	III–IV

HD, healthy donor; KOA, knee osteoarthritis; BMI, body mass index

### PBMC extraction and cell culture

The collected peripheral blood was diluted with an equal volume of phosphate-buffered saline (PBS). A 15-ml centrifuge tube with 5 ml of Ficoll Separation Solution (Ficoll-Paque PREMIUM, Cytiva) was prepared. The peripheral blood was slowly added to the centrifuge tube to keep the liquid levels separate and centrifuged at 2,000 rpm for 20 min, maintaining a gentle rise and fall during centrifugation. PBMCs in the middle layer was collected at the end of centrifugation, resuspended in PBS to wash the Ficoll separation solution, and centrifuged again to obtain PBMCs. The EasySep Human Monocyte Isolation Kit & EasySep Mouse Monocyte Isolation Kit (Stemcell) were used according to the reagent manufacturer’s instructions to magnetically sort PBMCs into monocytes. After washing with PBS, PBMCs were added to RPMI 1640 medium (61870036, Gibco) in a 37 °C, 5% CO_2_ environment. THP-1 cells were obtained from Zhejiang BioDee Biotechnology Co., Ltd. (C5157), and THP-1 cells were cultured under the same conditions as primary monocytes.

### Flow cytometry and sorting

The separated mononuclear cells were diluted in fluorescence-activated cell sorting (FACS) buffer (PF00018, Proteintech), Fc receptor blocking antibodies (BD Pharmingen Purified NA/LE Human BD Fc Block & Mouse BD Fc Block, BD) were added and incubated in an ice bath for 30 min. Then, they were diluted in FACS buffer, and FITC-CXCR4 (CXCR4-401AP, Fabgennix) was added. The antibodies were incubated for 30 min in the dark. After incubation, cells are washed 3 times and measured on a FACS Calibur (BD, Franklin Lakes, New Jersey). For flow cytometry, cells were washed with PBS and stained with Hoechst 33342 to collect viable cells. Cells were sorted using a BD FACSAria III, and CXCR4^+^ macrophages were selected by plotting FSC-A and FSC-W, and viable cells were selected by Hoechst 33342. Cells were resuspended in 10% FBS Dulbecco’s modified Eagle medium and collected by centrifugation.

### Experimental design

In the in vivo experiment, HMGB1 neutralizing antibody (Nab-HMGB1, SQab20175, China Arigo Biological Company) was injected into the tail vein at a dose of 10 μg/d throughout the intervention process. For the CXCR4 inhibitor intervention, mice were injected with plerixafor (HY-10046, MCE, USA) at a dose of 5 mg/kg into the tail vein.

In the in vitro experiment, HMGB1 intervention was performed by treating the cells with 40 ng/ml recombinant HMGB1 protein (HY-P70570, MCE, USA); CCL4 intervention was performed by treating the cells with 50 ng/ml CCL4 (HY-P7257, MCE, USA); for IL-18 intervention, 1 ng/ml IL-18 (HY-P70591, MCE, USA) was used to treat the cells.

### Animal fluorescence in vivo imaging

CXCR4^+^ monocytes after flow sorting were counted using a Countess II automated cell counter to ensure that the cell count was 5 × 10^5^ per infusion and labeled with 5 μM DiR fluorescent dye (HY-D1048, MCE, USA). Then, after tail vein injection, the migration site of CXCR4^+^ monocytes was observed using VISQUE InVivo Smart-LF (Shanghai Biotime Biotech Co., Ltd.).

### Histopathology

The collected synovial tissues were fixed in 4% paraformaldehyde for 12 h, washed with PBS, embedded in paraffin, and serially sectioned. The sections were stained with HE (C0105S, Beyotime Biotechnology) and MASSON (C0189S, Beyotime Biotechnology) kits according to the manufacturer’s instructions. Specific methods are described above.

### ELISA array

This study utilized a customized multifactor microarray ELISA developed by Wuhan ABclonal to detect cytokine levels in the samples. These 15 selected cytokines, including CCL2, CCL3/MIP-1α, CCL4/MIP-1β, CXCL8, CXCL12, CXCL16, IL-1β, IL-2, IL-6, IL-18, IL-33, TNF-α, HMGB1, S100A8, and S100A9, were used to observe inflammation levels. To minimize data bias during the assay, each sample was measured 3 times, and the average of these values was calculated. A standard curve was used to quantify the cytokine concentrations. OD (optical density) values were determined using a PerkinElemer EnVision microplate reader and cytokine concentrations were determined based on the standard curve.

### Western blotting and coimmunoprecipitation

Sample protein was extracted using radioimmunoprecipitation assay lysis buffer, and the amount of protein was loaded onto a sodium dodecyl sulfate-polyacrylamide gel for electrophoresis. After electrophoresis, it was transferred to a polyvinylidene fluoride membrane and blocked with 5% skim milk for 1 h, and the membrane was washed with tris-buffered saline with Tween 20 (TBST) for 30 min. Glyceraldehyde-3-phosphate dehydrogenase (GAPDH) (Abcam, 8245, 1:1,000), IL-1β (Abcam, # 283818, 1:1,000), IL-6 (Thermo Fisher Scientific, # 701028, 1:1,000), TNF-α (Abcam, # 183218, 1:1,000), IRF1 (CST, # 8478, 1:1,000), and MYD88 (Abcam, # 219413, 1:1,000) were added and incubated. The next day, the membrane was washed with TBST for 30 min and then incubated with the appropriate secondary antibodies; anti-mouse immunoglobulin G (IgG), horseradish peroxidase-conjugated antibody (CST, #7076, 1:2,000), and anti-rabbit IgG, horseradish peroxidase-conjugated antibody (CST, #7074, 1:2,000) were incubated at room temperature for 1 h, and the membrane was washed with TBST for 30 min. The protein was then exposed and stained with enhanced chemiluminescence reagent, and the target band was analyzed using ImageJ for gray value calculation.

For coimmunoprecipitation, the protein lysates of monocytes and THP-1 cells were immunoprecipitated with immunoprecipitation buffer containing agarose beads coupled with specific immunoprecipitation antibodies, and the protein complexes obtained by coprecipitation were detected by Western blot. The subsequent detection steps were the same as described above. In addition to the above primary antibodies, the following antibodies were used Sumo 1 (Abcam, #32058, 1:1,000), Sumo 2/3 (Proteintech, #11251-1-AP, 1:1,000), and TRIM28 (Abcam, #109287, 1:1,000), IRF1 (Abcam, #247408, 1:1,000). IgG was used as NC.

### Real-time PCR

Determination of *IL1B*, *IL6*, and *TNFA* mRNA expression levels in the sample. Total mRNA was extracted from each sample using TRIzol reagent, and cDNA was obtained by reverse transcription according to the instructions of the reverse transcription kit. The reverse transcribed cDNA was used as a template, and primers and probes were added to perform a real-time polymerase chain reaction (PCR). Finally, *GAPDH* was used as an internal reference, and the relative expression of the target gene was calculated by 2^−△△CT^. The specific primer sequences are shown in Tables 2 and 3.

**Table 2.** Primers for real-time PCR (human)

Name	Description	Primer (5′-3′)
*IL1B*	Forward	TGGCCAAGCCTCAGACCT
	Reverse	GGTTCAGCTCGGCTTGTG
*IL6*	Forward	CCTCTCTGCAAGAGACTTCC
	Reverse	CAGGCAATGAGAAGTCTCCT
*TNFA*	Forward	GGTCCCTCAGACCCTCAG
	Reverse	GCTACGACGTGGGCTG
GAPDH	Forward	GAGTCAACGGATTTGGTCGT
	Reverse	TTGATTTTGGAGGGATCTCG

GAPDH, glyceraldehyde-3-phosphate dehydrogenase; PCR, polymerase chain reaction

**Table 3.** Primers for real-time PCR (mouse)

Name	Description	Primer (5′-3′)
*Il1b*	Forward	GAAATGCCACCTTTTGACAGTG
	Reverse	TGGATGCTCTCATCAGGACAG
*Il6*	Forward	CCACTTCACAAGTCGGAGGCTTA
	Reverse	GCAAGTGCATCATCGTTGTTCATAC
*Tnfa*	Forward	GACCCTCACACTCAGATCATCTTCT
	Reverse	CCACTTGGTGGTTTGCTACGAC
GAPDH	Forward	TGTGTCCGTCGTGGATCTGA
	Reverse	TTGCTGTTGAAGTCGCAGGAG

GAPDH, glyceraldehyde-3-phosphate dehydrogenase; PCR, polymerase chain reaction

### Immunofluorescence

The cells or paraffin sections fixed with 4% paraformaldehyde were permeabilized with Triton X-100 (P0096, Beyotime Biotechnology), blocked with 5% bovine serum albumin, and then incubated with primary or secondary antibodies, respectively. The slides were finally sealed with anti-fluorescence quenching sealing solution containing 4′,6-diamidino-2-phenylindole (P0131, Beyotime Biotechnology). The cell or tissue paraffin sections were observed by fluorescence microscopy or laser confocal microscopy.

Primary antibodies used were as follows: IRF1 (CST, # 8478, 1:100), MyD88 (Proteintech, # 67969-1-Ig, 1:200), CXCR4 (Abcam, # 181020, 1:500), CD11b (Abcam, # 8878, 1:50), and LY6C (Abcam, # 317272, 1:500). Secondary antibodies: Alexa Fluor 488 goat anti-rabbit (Thermo Fisher Scientific # A-11008, 1:500), Alexa Fluor 594 goat anti-mouse (Thermo Fisher Scientific # A-11005, 1:500), and Alexa Fluor 647 goat anti-mouse (Thermo Fisher Scientific # A-21245, 1:500).

### Bulk transcriptomics (RNA-seq) and data analysis

Total RNA was extracted from each cell sample using TRIzol (Invitrogen, CA, USA). The purity and concentration of total RNA was then determined using a NanoDrop ND-1000 (NanoDrop, Wilmington, DE, USA). The integrity of the RNA was then tested using a Bioanalyzer 2100 (Agilent, CA, USA), and quality control was verified using an agarose gel electrophoresis protocol. It was ensured that the RNA concentration is >50 ng/μl, RIN is >7.0, OD260/280 is >1.8 and total RNA is >1 μg. Oligo(dT) magnetic beads (Dynabeads Oligo [dT], Cat. No. 25-61005, Thermo Fisher, USA) were used to specifically capture mRNA with PolyA (polyadenylate) through 2 rounds of purification. The captured mRNA was fragmented at high temperatures using a magnesium ion-dependent fragmentation reagent (NEBNext Magnesium RNA Fragmentation Module, Cat. No. E6150S, USA) for 5 to 7 min at 94 °C. The fragmented RNA was used for cDNA synthesis by reverse transcriptase (Invitrogen SuperScript II Reverse Transcriptase, Cat. No. 1896649, CA, USA). Double-strand synthesis was then performed using *E. coli* DNA Polymerase I (NEB, Cat. No. m0209, USA) and RNase H (NEB m0297, USA) to convert these DNA and RNA hybrid double strands into DNA double strands. At the same time, dUTP solution (Thermo Fisher, R0133, CA, USA) was incorporated into the double strand to complete the ends of the double-stranded DNA into flat ends. Then, an A base was added to each end to allow it to connect to a joint with a T base at the end, and magnetic beads were used to screen and purify the fragment size. The double strand was digested with the enzyme UDG (NEB, Cat. No. m0280, MA, US), and then a library was constructed by PCR. Finally, we used the Illumina NovaSeq 6000 (LC Bio Technology CO., Ltd. Hangzhou, China) for paired-end sequencing with a sequencing mode of PE150.

After sequencing, the raw data format was fastq. Raw data were quality-controlled using the fastp software (https://github.com/OpenGene/fastp), including removal of adapters, repetitive sequences, and low-quality sequences, with default parameters. The HISAT2 (https://ccb.jhu.edu/software/hisat2) was used to align the sequencing data to the genome (Homo sapiens, GRCh38), and the file format was bam. The StringTie software (https://ccb.jhu.edu/software/hisat2) was used to assemble genes or transcripts and quantify them using FPKM (FPKM = total_exon_fragments / mapped_reads (millions) × exon_length (kB)]), and the R package edgeR (https://bioconductor.org/packages/release/bioc/html/edgeR.html) was used to analyze differentially expressed genes between samples. A fold change of >2 or <0.5 and a *P* value <0.05 were defined as differentially expressed. Finally, GO and KEGG enrichment analysis of genes was performed using the DAVID software (https://davidbioinformatics.nih.gov/).

### Single-cell RNA-seq

The extracted mononuclear cells were resuspended in PBS to prepare a cell suspension. The cell suspension was filtered through a 70- to 30-μm cell sieve and centrifuged to collect the cell pellet, and 100 μl of Dead Cell Removal Microbeads (MACS 130-090-101) was added. Cell activity was then detected by the trypan blue staining method, and cell activity was required to be >85%. The cell number was counted using an automated cell counter (Countess II Automated Cell Counter) to ensure that it was 700 to 1,200 cells/μl.

The final cell suspension was loaded into a 10X Chromium instrument for cell capture, cDNA amplification, and library construction according to the instructions of the official library construction kit (10x Genomics Chromium Single-Cell 3’ kit, V3). After library construction, sequencing was performed on the NovaSeq 6000 sequencing platform (paired-end multiplex run, 150 bp) with a sequencing depth of 20,000 reads per cell.

### Single-cell RNA-seq data processing

Reads were processed using the Cell Ranger v3 pipeline with default and recommended parameters. FASTQs generated from Illumina sequencing output were aligned to the GRCh38 reference genome, using the STAR algorithm. Next, Gene-Barcode matrices were generated for each individual sample by counting UMIs and filtering noncell-associated barcodes. Finally, we generated a gene-barcode matrix containing the barcoded cells and gene expression counts. This output was imported into the Seurat (v5.3.0) R toolkit (R version 4.4.0) for quality control and downstream analysis for our single-cell RNA-seq datal. We first filtered the matrices to exclude low-quality cells following the next relatively-loose criteria: (a) number of detected transcripts (number of unique UMIs) was more than 200; (b) detected genes were between 100 and 10,000; and (c) percent of reads mapping to mitochondrial genes was no more than 25%. The expression percent of mitochondria genes was calculated using Percentage FeatureSet function of the Seurat R package. For required further analysis, NormalizeData was performed with default parameters. The FindVariableFeatures finction in Seuratpackage was performed for extracting a subset of variable genes (selection.method = “vst”, nfeatures = 2000). Then, we performed PCA with parameters of dims=20 after scaling the data with ScaleData function in Seurat using all detected genes as input features. Next, we integrated data from different samples using RunHarmony method in R package harmony (v1.2.3) with default parameters. We visualized the clusters on a 2D map produced with uniform manifold approximation and projection (UMAP), mainly with the DimPlot function in Seurat. [70]

### Identification of cell types and subtypes by UMAP

Cells were clustered using graph-based clustering of the PCA reduced data with the RunUMAP function. For subclustering, we used FindClusters function (resolution = 1) in Seurat. For each cluster, we used the Wilcoxon rank-sum test to find significant deferentially expressed genes comparing the remaining clusters (min.pct = .25, logfc.threshold = .25). Canonical markers for possible cell type was used for DotPlot in Seurat using slot “data”.

### Monocyte subclustering and exploration

Reclustering of all Monocytes was performed using the same workflow as former-mentioned, especially using also FindClusters function with resolution = 1. Similarity between clusters was evaluated by calculating jaccard similarity of each 2 cell groups, after selecting top 100 positive marker genes of each cluster as input. For further understanding of function for each cluster, gsva (v2.0.7) in R was used against the pseudo-bulk expression generated with AverageExpression (param: assays = ’RNA’, slot = ’data’) function in Seurat. Heatmap plot was performed with pheatmap (1.0.12) in R. For inferring the development trace of monocytes between 2 sample groups, we firstly execute CytoTRACE2 (1.0.2) to predict the stemness or potency of each subcluster. After that, Monocle2 (v2.34.0) in R, using monoce hvg (dispersion Table function in monocle V2) as input genes. The root site for monocle2 was determined according to the former cytoTRACE2 result. In order to find conservative biological function across individual samples or monocyte clusters, especially for the integrated cluster 3_7_10 which showed most activity of Inflammatory response, we performed NMF method to get stable MP (functional gene sets) with geneNMF (v0.8.0) in R package, with parameter of k_number=2:10 and min.exp=0.05 in multiNMF;metric = “cosine”,weight.explained =0.3, min.confidence = 0.3, nMP=4 in getMetaPrograms. Functional analysis and network visualization of all gene sets was performed with clusterProfiler (v4.14.6) and customized script of ggplot2 in R package (v3.5.2).

### Bulk ATAC-seq and data analysis

Cells were counted using an automated cell counter (Countess II Automated Cell Counter). After the cells were lysed for 5 min, the nuclei were collected, suspended in a transposition reaction system containing Tn5 transposase, and incubated; the DNA was purified, and then the product was amplified while introducing specific indices. The mixture was incubated at 72 °C for 3 min, predenatured at 98 °C for 1 min, denatured at 98 °C for 10 s, annealed at 60 °C for 25 s, and extended at 72 °C for 25 s for a total of 13 to 15 cycles. The extension was held at 72 °C for 5 min, and the mixture was sorted using magnetic beads, 1 min, 98 °C denaturation for 10 s, 60 °C annealing for 25 s, 72 °C extension for 25 s, a total of 13 to 15 cycles, 72 °C extension retention for 5 min, and the library fragments of approximately 200 to 700 bp were obtained by magnetic bead sorting. The concentration of the library was detected using Qubit (Thermo, Qubit 3.0, USA), and the integrity of the fragments was checked using a Bioanalyzer 2100 (Agilent, CA, USA). PE150 sequencing was performed using Illumina NovaSeq XP according to standard operating procedures.

The fastp software was used to remove adapter sequences, low-quality bases, and filter low-quality and short fragments from the raw reads. CleanData was used for subsequent genome alignment, peak detection, and other analyses. The bowtie2 software was used to align the reads to the reference genome, and the alignment results were saved in SAM or BAM file format. After alignment, the data were further processed to remove duplicates, filter low-quality alignment sequences, and exclude mitochondrial DNA. The MACS2 software was used to identify peak regions, and the PeakCalling results were annotated using the ChIPseeker software to annotate downstream functional regions of the genome. For projects with biological replicates, the diffbind software was used to perform differential analysis of peaks, while for projects without biological replicates, the MAnorm software was used for differential analysis. The homer software was used to perform motif analysis of differential peak results, and hypergeometric testing was used to perform GO/KEGG enrichment analysis of genes downstream of differential peaks.

### Statistical analysis

Data are presented as bar graphs with error bars and line graphs (mean ± standard error of the mean [SEM]). All statistical analyses were performed with GraphPad Prism 10. All relevant datasets (including fold changes and percentages) were log-transformed to normalize the distribution. Statistical analyses of 2 groups were performed using a paired 2-tailed *t* test or an unpaired 2-tailed *t* test (with Welch correction), as appropriate. Statistical analysis of comparisons between multiple groups was performed using 1- or 2-way analysis of variance (ANOVA). A 2-tailed test was used for all comparisons, and a single *P* or false discovery rate-corrected *P* (*q*) < 0.05 was considered statistically significant.

## Ethical Approval

The animal experiments in this study were approved by the Animal Ethics Committee of Nanjing University of Chinese Medicine (Approval No: 2024NL-KS095). The source of clinical samples was approved by the Medical Ethics Committee of the Affiliated Hospital of Nanjing University of Chinese Medicine (Approval No: 2022NL-180-01).

## Data Availability

All sequencing data have been uploaded to the NCBI public database. Clinical PBMC single-cell transcriptome sequencing: PRJNA1273613; clinical PBMC ATAC-seq: PRJNA1276062; ATAC-seq of PBMCs from mice treated with either negative control or HMGB1: PRJNA1276150; Bulk RNA-seq of PBMCs from mice treated with either negative control or HMGB1: PRJNA1275555; Bulk RNA-seq of PBMCs from KOA mice at different time points: PRJNA1275461; and bulk RNA-seq of CXCR4^+^ and CXCR4− PBMCs from mice: PRJNA1273625. All results and data are maintained in the Department of Orthopedics, the Affiliated Hospital of Nanjing University of Chinese Medicine, Nanjing, China. All data supporting the findings of this manuscript are also available from the corresponding authors upon request. This includes deidentified participant data referenced in the manuscript, as well as the study protocol and materials related to ethical approval.
