# Supplementary material for: High Mobility Group Protein B1 Promotes Interferon Regulatory Factor 1 SUMOylation to Prime Trained Immunity of Circulating Monocytes and Aggravate the Progressive Synovial Inflammation in Knee Osteoarthritis
Source: Research (Wash D C). 2026 May 14;9:1243. doi: 10.34133/research.1243 (PMC13172585; doi:10.34133/research.1243)
Supplement: Supplementary 1 — Tables S1 and S2 Figs. S1 to S8 [file research.1243.f1.zip › Supplementary information.docx]

**METHODS**

**Laboratory animals and modeling**

Wild-type C57BL/6J male mice (6-8 weeks old) were purchased from Jiangsu Huanoxinchuang Co, Ltd, and MYD88 knockout (KO) C57BL/6J mice (6-8 weeks old) were purchased from Jiangsu Jicui Yaokang Biotechnology Co, Ltd. The animals were maintained in an SPF animal room with a temperature of 26±0.5°C, humidity of 50±5%, and a 12-hour light-dark cycle, with free access to water and food. We used 1 mg iodoacetic acid (I104613, Shanghai Aladdin Biochemical Technology Co., Ltd.) to establish a KOA model by knee joint injection using a microsyringe (Hamilton), and stimulation was performed on days 0 and 28. This animal experiment was approved by the Animal Ethics Committee of Nanjing University of Traditional Chinese Medicine (Approval number: 2024NL-KS095).

**Clinical sample collection**

The peripheral blood mononuclear cell study was approved by the Medical Ethics Committee of the Jiangsu Provincial Hospital of Traditional Chinese Medicine (approval number: 2022NL-180-01). Patients with KOA (n = 6, male) and healthy volunteers (HD) (n = 6, male) were recruited from the Jiangsu Provincial Hospital of Traditional Chinese Medicine, and peripheral blood mononuclear cells were obtained by peripheral blood collection. Exclusion criteria included a history of diabetes, arterial hypertension, chronic renal failure, signs or history of infection at the time of inclusion, or any history or clinical symptoms of inflammatory disease. It was also ensured that the HD had no history of back pain, arthritis, joint damage or other inflammation. Written informed consent was obtained from all participants.

**Peripheral blood mononuclear cell extraction and cell culture**

Dilute the collected peripheral blood with an equal volume of PBS. Prepare a 15 mL centrifuge tube with 5 mL of Ficoll Separation Solution (Ficoll-Paque™ PREMIUM, Cytiva). Slowly add the peripheral blood to the centrifuge tube to keep the liquid levels separate. Centrifuge at 2,000 rpm for 20 minutes, maintaining a gentle rise and fall during centrifugation. Collect peripheral blood mononuclear cells (PBMCs) in the middle layer at the end of centrifugation, resuspend in PBS to wash the Ficoll separation solution, and centrifuge again to obtain PBMCs. Use the EasySep™ Human Monocyte Isolation Kit & EasySep™ Mouse Monocyte Isolation Kit (Stemcell) according to the reagent manufacturer's instructions to magnetically sort PBMCs into monocytes. After washing with PBS, PBMCs were added to Roswell Park Memorial Institute (RPMI) 1640 medium (61870036, Gibco) in a 37°C, 5% CO2 environment. THP-1 cells were obtained from Zhejiang BioDee Biotechnology Co., Ltd. (C5157), and THP-1 cells were cultured under the same conditions as primary monocytes.

**Flow cytometry and Sorting**

The separated mononuclear cells were diluted in FACS buffer (PF00018, Proteintech), Fc receptor blocking antibodies (BD Pharmingen™ Purified NA/LE Human BD Fc Block™ & Mouse BD Fc Block™, BD) were added and incubated in an ice bath for 30 minutes. Then dilute in FACS buffer and add APC-CXCR4 (146507, BioLegend). Incubate for 30 minutes in the dark. After incubation, cells are washed three times and measured on a FACS Calibur (BD, Franklin Lakes, New Jersey). For flow cytometry, cells were washed with PBS and stained with Hoechst 33342 to collect viable cells. Cells were sorted using a BD FACSAria™ III, and CXCR4+ macrophages were selected by plotting FSC-A and FSC-W, and viable cells were selected by hoechst 33342. Cells were resuspended in 10% FBS DMEM medium and collected by centrifugation.

**Experimental Design** ­

In the in vivo experiment, HMGB1 neutralizing antibody (Nab-HMGB1, SQab20175, China Arigo Biological Company) was injected into the tail vein at a dose of 10 μg/day throughout the intervention process. For the CXCR4 inhibitor intervention, mice were injected with plerixafor (HY-10046, MCE, USA) at a dose of 5 mg/kg into the tail vein.

In the in vitro experiment, HMGB1 intervention was performed by treating the cells with 40 ng/mL recombinant HMGB1 protein (HY-P70570, MCE, USA); CXCL12 intervention was performed by treating the cells with 50 ng/mL CXCL12 (HY-P7285, MCE, USA); for IL-18 intervention, 1ng/mL IL-18 (HY-P70591, MCE, USA) was used to treat the cells.

**Animal fluorescence in vivo imaging**

CXCR4+ monocytes after flow sorting were counted using a Countess II automated cell counter to ensure that the cell count was 5 x 105 per infusion and labeled with 5 μM DiR fluorescent dye (HY-D1048, MCE, USA). Then, after tail vein injection, the migration site of CXCR4+ monocytes was observed using VISQUE InVivo Smart-LF (Shanghai Biotime Biotech Co., Ltd.).

**Histopathology**

The collected synovial tissues were fixed in 4% paraformaldehyde for 12 hours, washed with PBS, embedded in paraffin, and serially sectioned. The sections were stained with H&E (C0105S, Beyotime Biotechnology) and MASSON (C0189S, Beyotime Biotechnology) kits according to the manufacturer's instructions. Specific methods are described above.

**ELISA ARRAY**

The enzyme-linked immunosorbent assay (ELISA) was used to determine the level of cytokines in the sample. These 15 selected cytokines (CCL3/MIP-1α, CCL4/MIP-1 β, CXCL8, CXCL12, CXCL16, IL-1β, IL-2, IL-6, IL-18, TNF-α, HMGB1, S100A8, S100A9). To minimize data bias during the assay, each sample was measured three times and the average of these values was calculated. A standard curve was used to quantify the cytokine concentrations. OD values were determined using a PerkinElemer EnVision microplate reader and cytokine concentrations were determined based on the standard curve.

**WB and CO-IP**

Sample protein was extracted using RIPA lysis buffer, and the amount of protein was loaded onto an SDS-polyacrylamide gel for electrophoresis. After electrophoresis, it was transferred to a PVDF membrane, blocked with 5% skim milk for 1 hour, and the membrane was washed with TBST for 30 minutes. GAPDH (Abcam, 8245, 1:1000), IL-1β (Abcam, # 283818, 1:100 0), IL-6 (Thermo Fisher Scientific, # 701028, 1:1000), TNF-α (Abcam, # 183218, 1:1000), IRF1 (CST, # 8478, 1:1000), and MYD88 (Abcam, # 219413, 1:1000) were added and incubated. The next day, the membrane was washed with TBST for 30 minutes and then incubated with the appropriate secondary antibodies, anti-mouse IgG, HRP-conjugated antibody (CST, #7076, 1:2000) and anti-rabbit IgG, HRP-conjugated antibody (CST, #7074, 1:2000) were incubated at room temperature for 1 hour and the membrane was washed with TBST for 30 minutes. The protein was then exposed and stained with ECL reagent, and the target band was analyzed using ImageJ for gray value calculation.

For CO-IP, the protein lysates of monocytes and THP-1 cells were immunoprecipitated with IP buffer containing agarose beads coupled with specific IP antibodies, and the protein complexes obtained by co-precipitation were detected by Western blot. The subsequent detection steps were the same as described above. In addition to the above primary antibodies, the following antibodies were used Sumo 1 (Abcam, #32058, 1:1000), Sumo 2/3 (Proteintech, #11251-1-AP, 1:100 0), TRIM28 (Abcam, #109287, 1:1000), IRF1 (Abcam, #247408, 1:1000). IgG was used as negative control.

**Real-time PCR**

Determination of IL-1β, IL-6, and TNF-α mRNA expression levels in the sample Total mRNA was extracted from each sample using Trizol reagent, and cDNA was obtained by reverse transcription according to the instructions of the reverse transcription kit. The reverse transcribed cDNA was used as a template, and primers and probes were added to perform a real-time PCR reaction. Finally, GAPDH was used as an internal reference, and the relative expression of the target gene was calculated by 2^-△△CT^.

Table 1 Primers for real-time PCR (human)

| Name | Description | Primer (5′-3′) |
| --- | --- | --- |
| IL-1β | Forward | TGGCCAAGCCTCAGACCT |
|  | Reverse | GGTTCAGCTCGGCTTGTG |
| IL-6 | Forward | CCTCTCTGCAAGAGACTTCC |
|  | Reverse | CAGGCAATGAGAAGTCTCCT |
| TNF-α | Forward | GGTCCCTCAGACCCTCAG |
|  | Reverse | GCTACGACGTGGGCTG |
| GAPDH | Forward | GAGTCAACGGATTTGGTCGT |
|  | Reverse | TTGATTTTGGAGGGATCTCG |

Table 2 Primers for real-time PCR（mouse）

| Name | Description | Primer (5′-3′) |
| --- | --- | --- |
| IL-1β | Forward | GAAATGCCACCTTTTGACAGTG |
|  | Reverse | TGGATGCTCTCATCAGGACAG |
| IL-6 | Forward | CCACTTCACAAGTCGGAGGCTTA |
|  | Reverse | GCAAGTGCATCATCGTTGTTCATAC |
| TNF-α | Forward | GACCCTCACACTCAGATCATCTTCT |
|  | Reverse | CCACTTGGTGGTTTGCTACGAC |
| GAPDH | Forward | TGTGTCCGTCGTGGATCTGA |
|  | Reverse | TTGCTGTTGAAGTCGCAGGAG |

**Immunofluorescence**

The cells or paraffin sections fixed with 4% paraformaldehyde were permeabilized with Triton X-100 (P0096, Beyotime Biotechnology), blocked with 5% BSA, and then incubated with primary or secondary antibodies, respectively. The slides were finally sealed with anti-fluorescence quenching sealing solution containing DAPI (P0131, Beyotime Biotechnology). The cell or tissue paraffin sections were observed by fluorescence microscopy or laser confocal microscopy.

Primary antibodies used: IRF1 (CST, # 8478, 1:100), MYD88 (Proteintech, # 67969-1-Ig, 1:200), CXCR4 (Abcam, # 181020, 1:500), CD11b (Abcam, # 8878, 1:50), Ly6c (Abcam, # 317272, 1:500). Secondary antibodies: Alexa Fluor™ 488 goat anti-rabbit (Thermo Fisher Scientific # A-11008, 1:500), Alexa Fluor™ 594 goat anti-mouse (Thermo Fisher Scientific # A-11005, 1:500), Alexa Fluor™ 647 goat anti-mouse (Thermo Fisher Scientific # A-21245, 1:500).

**Bulk transcriptomics (RNA-seq) and data analysis**

Total RNA was extracted from each cell sample using TRIzol (Invitrogen, CA, USA). The purity and concentration of total RNA was then determined using a NanoDrop ND-1000 (NanoDrop, Wilmington, DE, USA). The integrity of the RNA was then tested using a Bioanalyzer 2100 (Agilent, CA, USA), and quality control was verified using an agarose gel electrophoresis protocol. Ensure that the RNA concentration is >50 ng/μL, RIN is >7.0, OD260/280 is >1.8 and total RNA is >1 μg. Use oligo(dT) magnetic beads (Dynabeads Oligo (dT), Cat. No. 25-61005, Thermo Fisher, USA) were used to specifically capture mRNA with PolyA (polyadenylate) through two rounds of purification. The captured mRNA was fragmented at high temperatures using a magnesium ion-dependent fragmentation reagent (NEBNext® Magnesium RNA Fragmentation Module, Cat. No. E6150S, USA) for 5-7 minutes at 94°C. The fragmented RNA was used for cDNA synthesis by reverse transcriptase (Invitrogen SuperScript™ II Reverse Transcriptase, Cat. No. 1896649, CA, USA). Double-strand synthesis is then performed using E. coli DNA Polymerase I (NEB, Cat. No. m0209, USA) and RNase H (NEB m0297, USA) to convert these DNA and RNA hybrid double strands into DNA double strands. At the same time, dUTP solution (Thermo Fisher, R0133, CA, USA) is incorporated into the double strand to complete the ends of the double-stranded DNA into flat ends. Then, an A base was added to each end to allow it to connect to a joint with a T base at the end, and magnetic beads were used to screen and purify the fragment size. The double strand was digested with the enzyme UDG (NEB, Cat. No. m0280, MA, US), and then a library was constructed by PCR. Finally, we used the Illumina Novaseq™ 6000 (LC Bio Technology CO., Ltd. Hangzhou, China) for paired-end sequencing with a sequencing mode of PE150.

After sequencing, the raw data format was fastq. Raw data were quality controlled using the fastp software (https://github.com/OpenGene/fastp), including removal of adapters, repetitive sequences, and low-quality sequences, with default parameters. The HISAT2 (https://ccb.jhu.edu/software/hisat2) was used to align the sequencing data to the genome (Homo sapiens, GRCh38) and the file format was bam. The StringTie software (https://ccb.jhu.edu/software/hisat2) was used to assemble genes or transcripts and quantify them using FPKM ( FPKM = total_exon_fragments / mapped_reads(millions) × exon_length(kB)]), and the R package edgeR (<https://bioconductor.org/packages/release/bioc/html/> edgeR.html) was used to analyze differentially expressed genes between samples. A fold change of >2 or <0.5 and a p-value <0.05 were defined as differentially expressed. Finally, GO and KEGG enrichment analysis of genes was performed using the DAVID software (https://david.ncifcrf.gov/).

**Single cell RNA sequencing (scRNA-seq)**

The extracted mononuclear cells were resuspended in PBS to prepare a cell suspension. The cell suspension was filtered through a 70-30 μm cell sieve, centrifuged to collect the cell pellet, and 100 μL Dead Cell Removal Microbeads (MACS 130-090-101) was added. Cell activity was then detected by the trypan blue staining method, and cell activity was required to be >85%. The cell number was counted using an automated cell counter (Countess II Automated Cell Counter) to ensure that it was 700-1200 cells/μL.

The final cell suspension was loaded into a 10X Chromium instrument for cell capture, cDNA amplification, and library construction according to the instructions of the official library construction kit (10X Genomics Chromium Single-Cell 3' kit ,V3). After library construction, sequencing was performed on the NovaSeq 6000 sequencing platform (paired-end multiplex run, 150bp) with a sequencing depth of 20,000 reads per cell.

**Single cell RNA-seq data processing**

Reads were processed using the Cell Ranger xx pipeline with default and recommended parameters. FASTQs generated from Illumina sequencing output were aligned to the GRCh38 reference genome, using the STAR algorithm. Next, Gene-Barcode matrices were generated for each individual sample by counting UMIs and filtering non-cell associated barcodes. Finally, we generated a gene-barcode matrix containing the barcoded cells and gene expression counts. This output was imported into the Seurat (v5.3.0) R toolkit(R version 4.4.0) for quality control and downstream analysis for our singlecell RNAseq datal. We first filtered the matrices to exclude low-quality cells following the next relatively-loose criteria: (1) nuber of detected transcripts (number of unique UMIs) was; (2)detected genes was between 100 and 10000 ; and (3) percent of reads mapping to mitochondrial genes was no more than 25%. The expression percent of mitochondria genes was calculated using PercentageFeatureSet function of the Seurat R package. For required further analysis, NormalizeData was performed with default parameters. The FindVariableFeatures finction in Seuratpackage was performed for extracting a subset of variable genes (selection.method = "vst", nfeatures = 2000). Then we performed principal component analysis(PCA) with parameters of dims=20 after scaling the data with ScaleData function in Seurat using all detected genes as input features. Next, we integrated data from different samples using RunHarmony method in R package harmony (v1.2.3) with default parameters. We visualized the clusters on a 2D map produced with UMAP, mainly with the DimPlot function in Seurat.

**Identification of cell types and subtypes by UMAP**

Cells were clustered using graph-based clustering of the PCA reduced data with the RunUMAP function. For sub-clustering, we used FindClusters function (resolution=1) in Seurat. For each cluster, we used the Wilcoxon Rank-Sum Test to find significant deferentially expressed genes comparing the remaining clusters(min.pct = .25, logfc.threshold = .25). Canonical markers for possible celltype was used for DotPlot in Seurat using slot ‘data’.

**Monocyte sub-clustering and exploration**

Re-clustering of all Monocytes was performed using the same workflow as former-mentioned, especially using also FindClusters funtion with resolution=1. Similarity between clusters was evaluated by calculating jaccard similarity of each two cell groups, after selecting top100 positive marker genes of each cluster as input. For further understanding of function for each cluster, gsva (v2.0.7) in R was used against the pseudu-bulk expression generated with AverageExpression(param: assays = 'RNA', slot = 'data') function in Seurat. Heatmap plot was performed with pheatmap (1.0.12) in R.For inferring the development trace of monocytes between two sample groups, we firstly execute CytoTRACE2(1.0.2) to predict the stemness or potency of each sub-cluster. After that, Monocle2(v2.34.0) in R, using monoce hvg(dispersionTable function in monocle V2) as input genes. The root site for monocle2 was determined according to the former cytoTRACE2 result. In order to find conservative biological function across individual samples or monocyte clusters, esecially for the integrated cluster 3_7_10 which showed most activity of Inflammatory response, we performed NMF method to get stable meta-programme (functional genesets) with geneNMF (v0.8.0) in R package, with parameter of k_number=2:10 and min.exp=0.05 in multiNMF;metric = "cosine",weight.explained =0.3, min.confidence = 0.3, nMP=4 in getMetaPrograms. Functional anaysis and network visualization of all genesets was performed with clusterProfiler (v4.14.6) and customized script of ggplot2 in R package (v3.5.2).

**Bulk assay for transposase-accessible chromatin with sequencing (ATAC-seq) and data analysis**

Cells were counted using an automated cell counter (Countess II Automated Cell Counter). After the cells were lysed for 5 min, the nuclei were collected, suspended in a transposition reaction system containing Tn5 transposase, incubated, and the DNA was purified, and then the product was amplified while introducing specific indices. The mixture was incubated at 72°C for 3 minutes, pre-denatured at 98°C for 1 minute, denatured at 98°C for 10 seconds, annealed at 60°C for 25 seconds, and extended at 72°C for 25 seconds for a total of 13 to 15 cycles. The extension was held at 72°C for 5 minutes and the mixture was sorted using magnetic beads. 1 min, 98 °C denaturation for 10 s, 60 °C annealing for 25 s, 72 °C extension for 25 s, a total of 13 to 15 cycles, 72 °C extension retention for 5 min, and the library fragments of approximately 200-700 bp were obtained by magnetic bead sorting. The concentration of the library was detected using Qubit (Thermo, Qubit 3.0, USA), and the integrity of the fragments was checked using a Bioanalyzer 2100 (Agilent, CA, USA). PE150 sequencing was performed using Illumina Novaseq XP according to standard operating procedures.

The fastp software was used to remove adapter sequences, low quality bases, and filter low quality and short fragments from the raw reads. CleanData was used for subsequent genome alignment, peak detection, and other analyses. The bowtie2 software was used to align the reads to the reference genome, and the alignment results were saved in SAM or BAM file format. After alignment, the data were further processed to remove duplicates, filter low quality alignment sequences, and exclude mitochondrial DNA. The MACS2 software was used to identify peak regions, and the PeakCalling results were annotated using the ChIPseeker software to annotate downstream functional regions of the genome. For projects with biological replicates, the diffbind software was used to perform differential analysis of peaks, while for projects without biological replicates, the MAnorm software was used for differential analysis. The homer software was used to perform motif analysis of differential peak results, and hypergeometric testing was used to perform GO/KEGG enrichment analysis of genes downstream of differential peaks.

**Statistical analysis**

Data are presented as bar graphs with error bars and line graphs (mean ± standard error of the mean (SEM)). All statistical analyses were performed with GraphPad Prism 10. All relevant data sets (including fold changes and percentages) were log-transformed to normalize the distribution. Statistical analyses of two groups were performed using a paired two-tailed t-test or an unpaired two-tailed t-test (with Welch correction), as appropriate. Statistical analysis of comparisons between multiple groups was performed using one- or two-way analysis of variance (ANOVA). A two-tailed test was used for all comparisons, and a single P or FDR-corrected P (q) < 0.05 was considered statistically significant.

**DATA AVAILABILITY**

All sequencing data have been uploaded to the NCBI public database. Clinical peripheral blood mononuclear cell single-cell transcriptome sequencing: PRJNA1273613; Clinical peripheral blood mononuclear cell ATAC-seq: PRJNA1276062; ATAC-seq of peripheral blood mononuclear cells from mice treated with either negative control or HMGB1: PRJNA1276150; Bulk RNA-seq of peripheral blood mononuclear cells from mice treated with either negative control or HMGB1: PRJNA1275555; Bulk RNA-seq of peripheral blood mononuclear cells from KOA mice at different time points: PRJNA1275461; and Bulk RNA-seq of CXCR4+ and CXCR4− peripheral blood mononuclear cells from mice: PRJNA1273625. All results and data are maintained in the Departments of Orthopedics, the Affiliated Hospital of Nanjing University of Chinese Medicine, Nanjing, China. All data supporting the findings of this manuscript are also available from the corresponding author upon request. This includes de-identified participant data referenced in the manuscript, as well as the study protocol and materials related to ethical approval.

**ACKNOWLEDGEMENTS**

The authors wish to express their gratitude to all staffs in the medical research center of first college of clinical medicine, the Nanjing University of Chinese Medicine, Nanjing, China.

**AUTHOR CONTRIBUTIONS**

Conception and design of research: Lishi Jie, Jun Mao, Li Zhang, Meng Cao, Peimin Wang. Performed experiments: Lishi Jie, Li Zhang, Houyu Fu, Taiyang Liao, Yibao Wei, Deren Liu, Jiaojiao Du. Analyzed data: Lishi Jie, Jun Mao, Li Zhang, Xiaochen Li, Peng Wu, Songjiang Yin, Nongshan Zhang. Interpreted results of experiments: Lishi Jie, Jun Mao, Li Zhang, Meng Cao, Peimin Wang.. Prepared figures: Lishi Jie, Jun Mao, Li Zhang, Meng Cao, Peimin Wang. Drafted manuscript: Lishi Jie, Jun Mao, Li Zhang, Meng Cao, Peimin Wang. Approved final version of manuscript: All authors.

**FUNDING**

This work was partly supported by Jiangsu Provincial Medical Key Discipline (Laboratory) Cultivation Unit (JSDW202252), Clinical Medical Innovation Center for Knee Osteoarthritis of TCM, Jiangsu Provincial Hospital of Chinese Medicine (Y2023zx05), Knee Osteoarthritis Specialized Clinical Research Institute, Nanjing University of Chinese Medicine (LCZBYJYZZ2024-003).

**COMPETING INTERESTS**

The authors declare no competing interests.


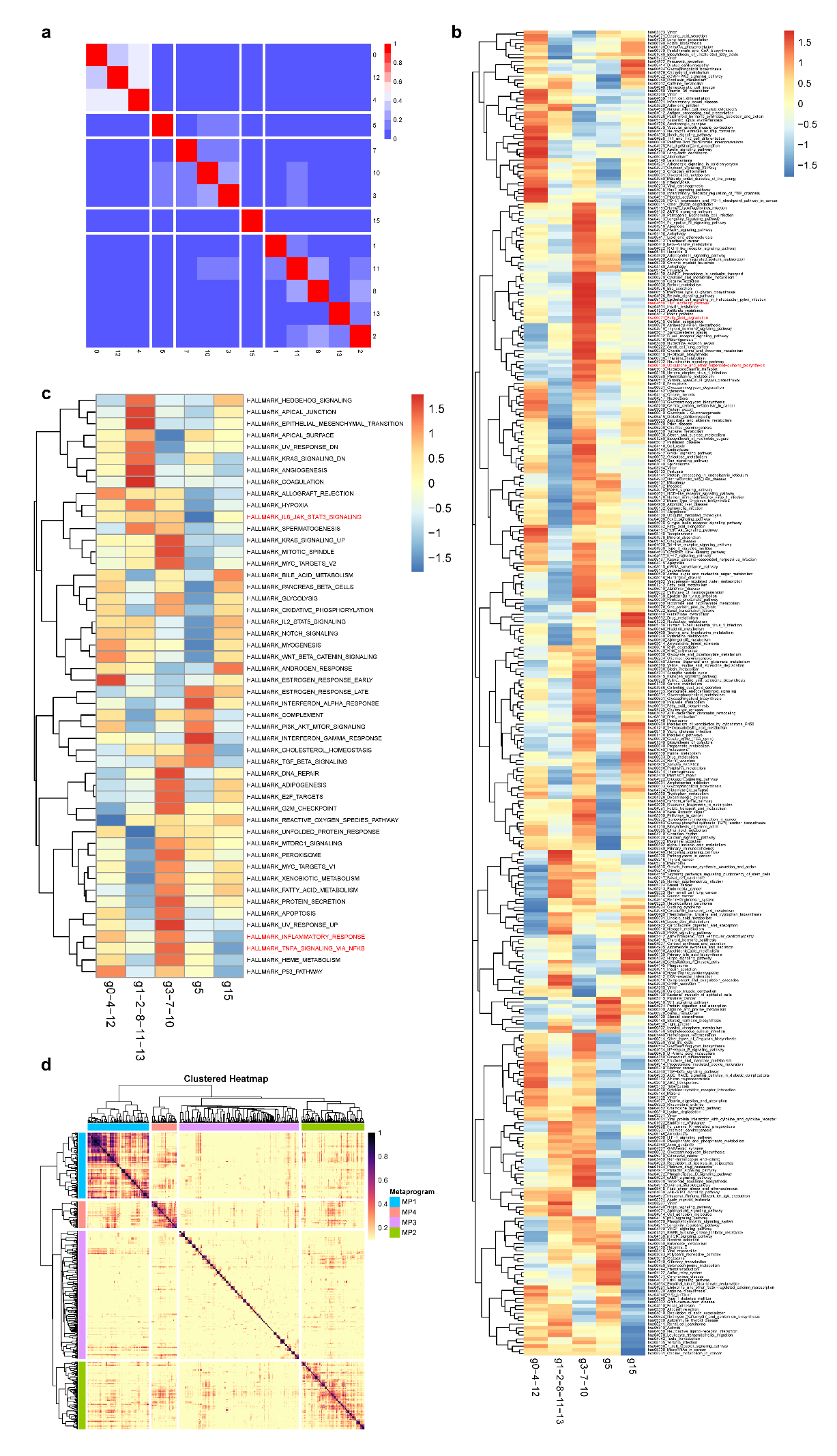


**S-Fig. 1: Single-cell transcriptomic analysis revealed the most pronounced inflammation-response-related signature among circulating monocyte.**

(a) The correlation heatmap of circulating monocyte subsets shows the correlations among each subset; (b) Enrichment analysis based on the KEGG database displays the top enriched pathways for each of the five functional groups; (c) Enrichment analysis based on the HALLMARK database presents the top enriched pathways for each of the five functional groups; (d) Non-negative matrix factorization was used to cluster genes from all cells in the g3-7-10 functional groups into four gene modules: MP1, MP2, MP3, and MP4, and a clustering heatmap was used to display the correlations within each gene module.


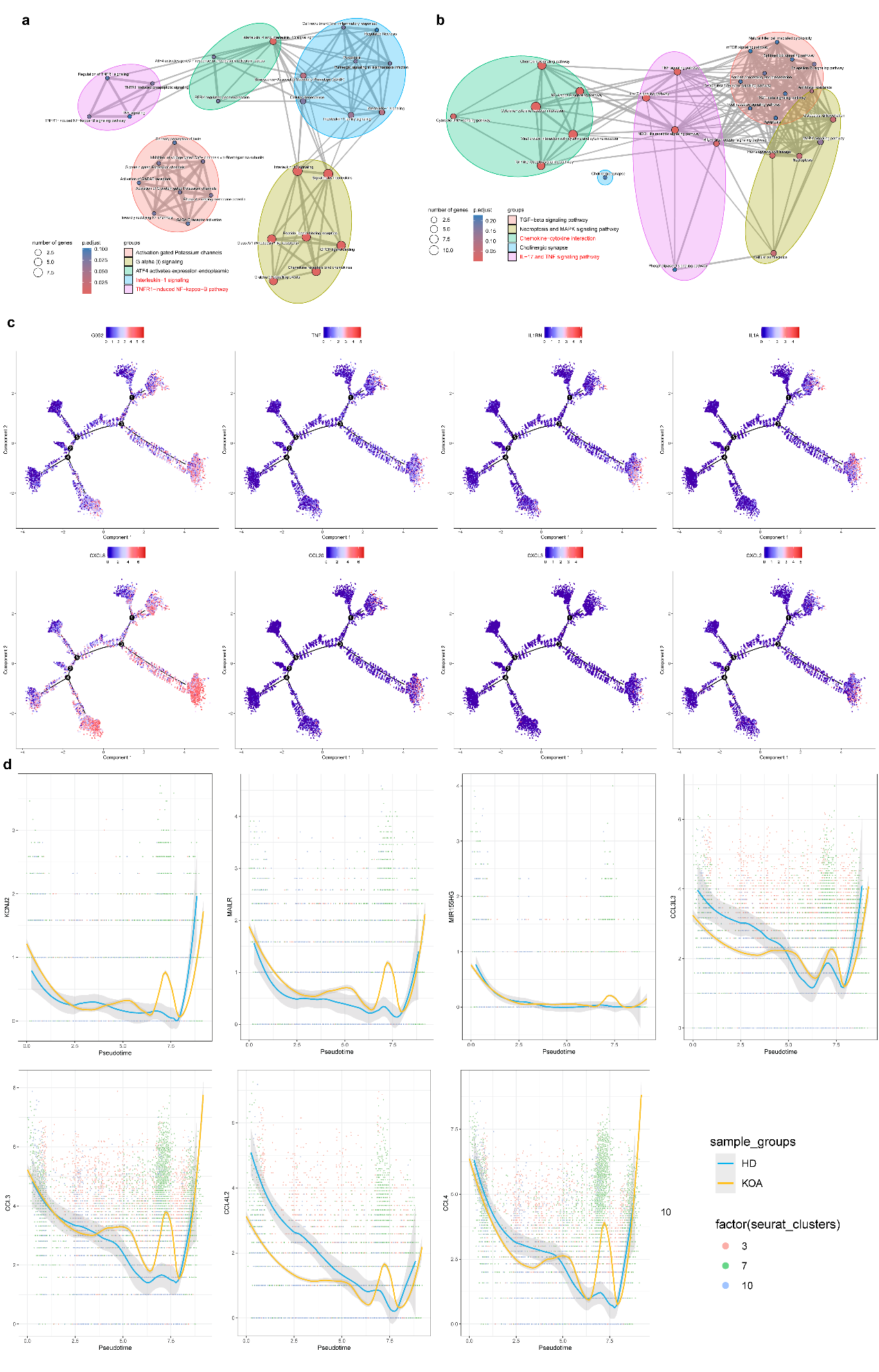


**S-Fig. 2: Characterization of circulating monocytes subpopulation g7 in KOA.**

(a) Network diagram generated by enrichment analysis of the M0P1 gene set based on the Reactome database; (b) Network diagram generated by enrichment analysis of the M0P1 gene set based on the KEGG database; (c) Distribution of IL1A, IL1R1, TNF, CXCL12, CXCL3, CXCL2, CCL20, and G0S2 from the MP1 gene set mapped onto the differentiation trajectory of the g3-7-10 functional clusters; (d) Distribution of genes KCNJ2, MAILR, MIR155HG, CCL3L3, CCL3, CCL4L2, and CCL4 from the MP1 gene set, which show no significant differences between the HD and KOA groups.


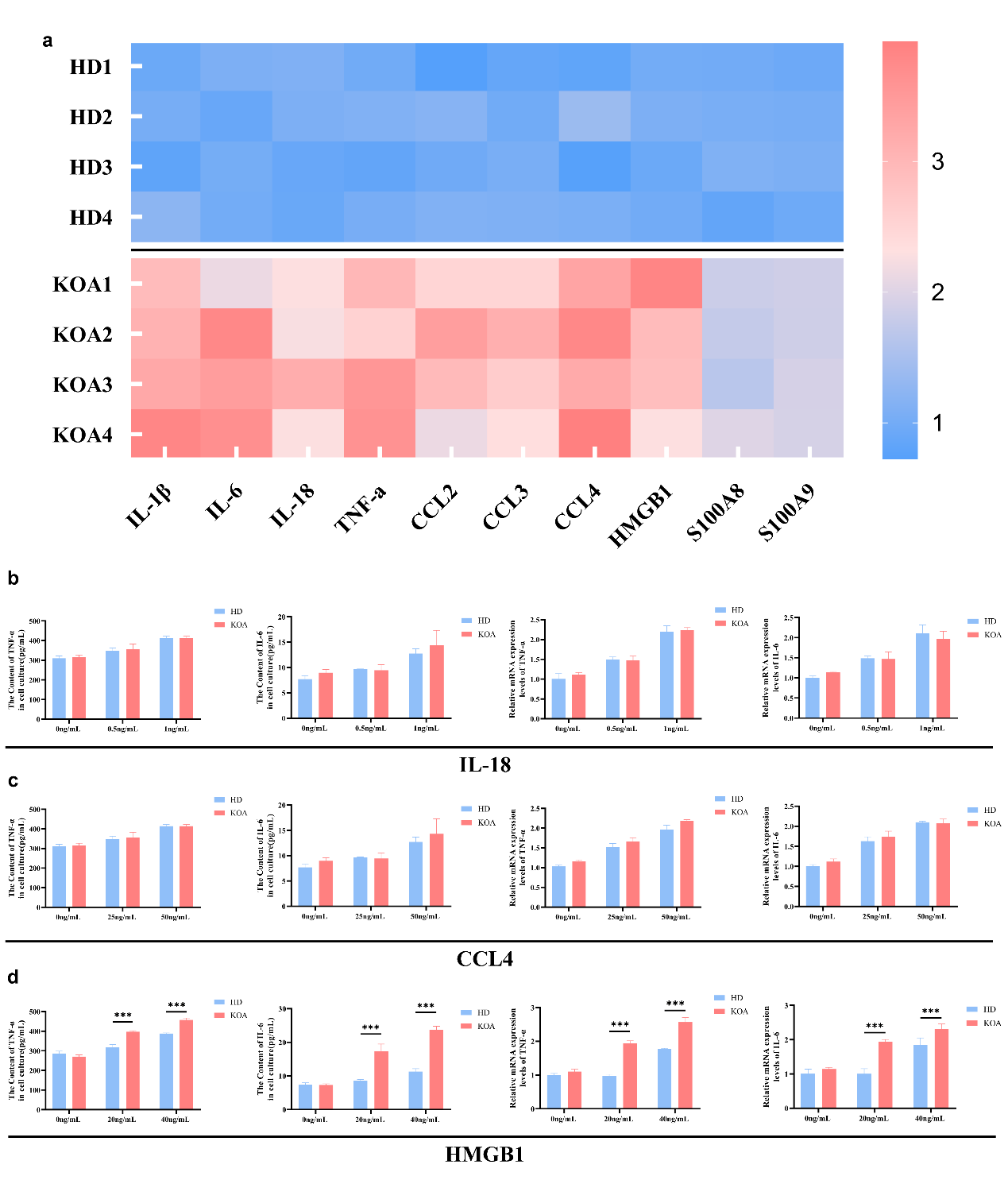


**S-Fig. 3: KOA-derived circulating monocytes undergo a trained phenotype.**

(a) Levels of inflammatory factors, chemokines and alertin family protein factors in peripheral serum of HD and KOA groups using array ELISA; (b) Protein and gene expression levels of TNF-α and IL-6 in peripheral circulating monocytes after IL-18 intervention using ELISA and qPCR; (c) Detection of protein and gene expression levels of TNF-α and IL-6 in peripheral circulating monocytes after CCL4 intervention using ELISA and qPCR; (d) Protein and gene expression levels of TNF-α and IL-6 in peripheral circulating monocytes after HMGB1 intervention using ELISA and qPCR. *statistical results were represented by mean ± standard error of the mean (Mean ±* SEM*), *P < 0.05, **P < 0.01,***P < 0.001.*


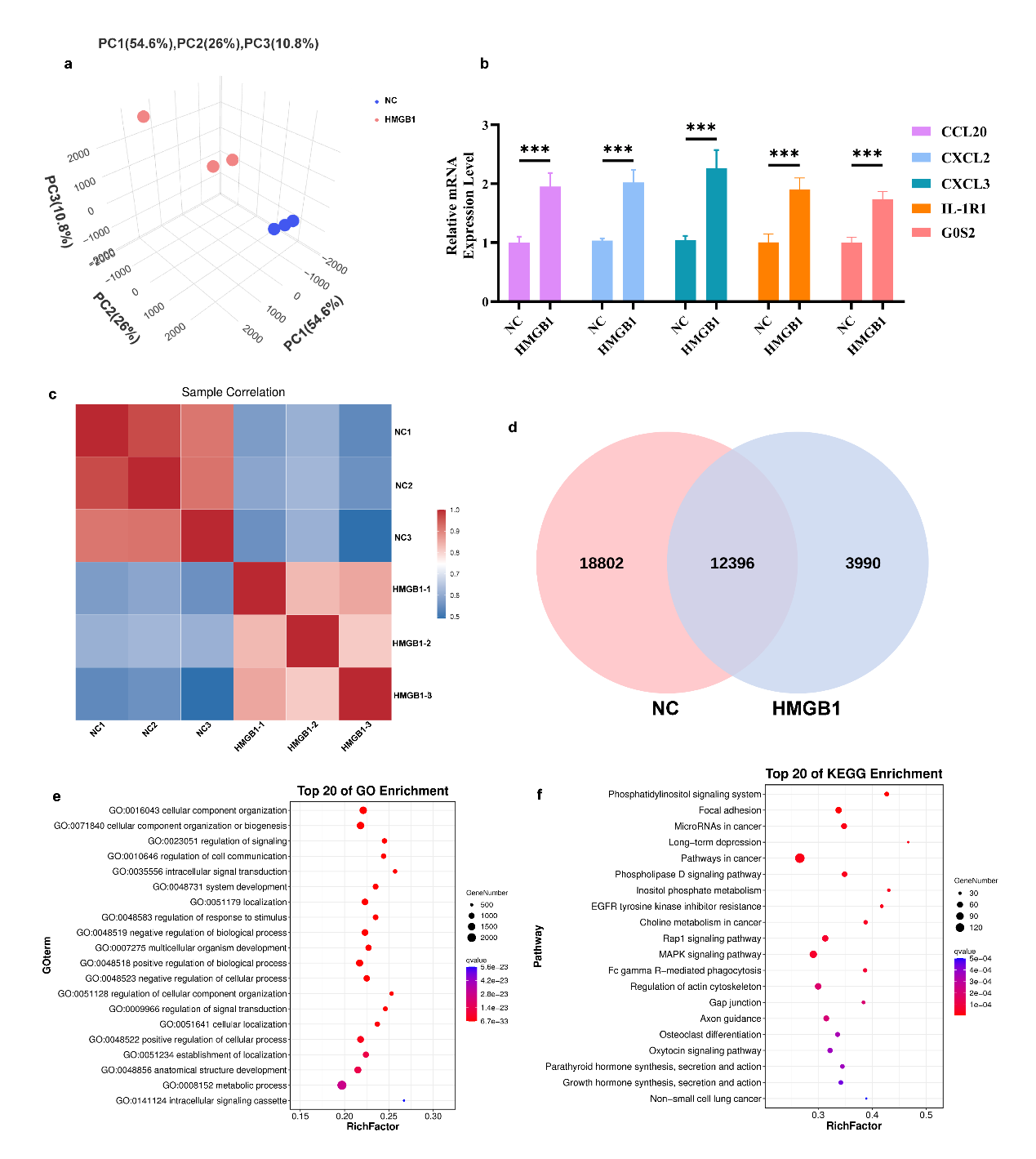


**S-Fig. 4: HMGB1 serves as a specific DAMP that stimulates circulating monocytes in KOA mice to adopt the g7 phenotype and exhibit features of trained immunity.**

(a) 3D PCA analysis shows a certain degree of separation in the spatial distribution of circulating monocytes between the NC group and the HMGB1 group in mice, indicating transcriptional differences; (b) PCR analysis measures the expression levels of IL1R1, CXCL3, CXCL2, CCL20, and G0S2 genes in circulating monocytes from the NC and HMGB1 groups; (c) Correlation heatmaps from ATAC-seq demonstrate differences in chromatin accessibility of circulating monocytes between the NC and HMGB1 groups; (d) Venn diagram shows the number of differentially accessible genes in circulating monocytes between the NC and HMGB1 groups; (e) GO database-based enrichment analysis of differentially expressed genes in circulating monocytes between the NC and HMGB1 groups; (f) KEGG database-based enrichment analysis of differentially expressed genes in circulating monocytes between the NC and HMGB1 groups.


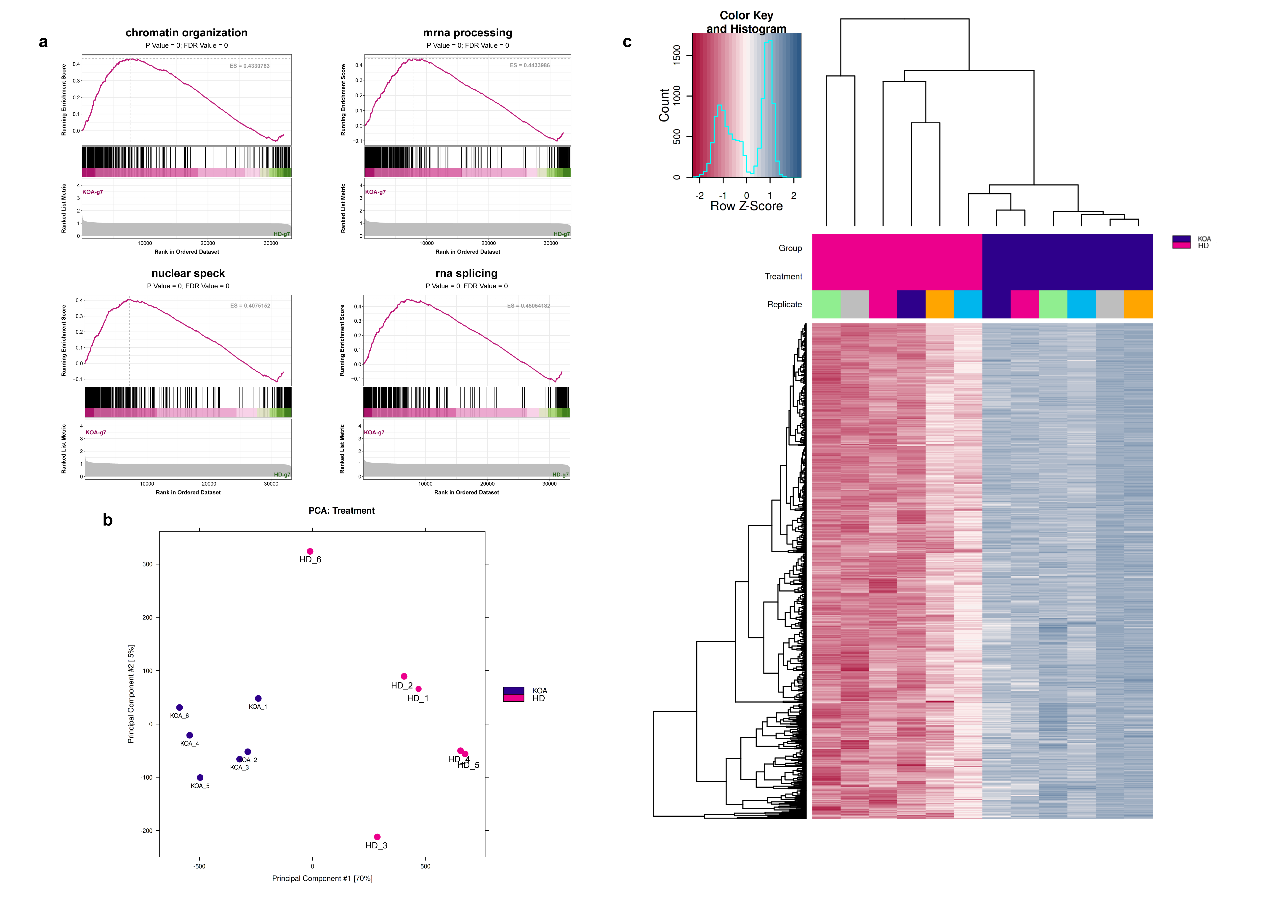


**S-Fig. 5: Trained circulating monocytes from KOA patients showed enhanced chromatin accessibility for the transcription factors IRF1, IRF3.**

(a) GSEA analysis based on the GO database revealed differences in the pathways “chromatin organization,” “mRNA processing,” “nuclear speck,” and “RNA splicing” in peripheral mononuclear cells of the “g7” subtype between the HD and KOA groups; (b) PCA analysis showed the chromatin accessibility levels in peripheral mononuclear cells from the HD and KOA groups; (c) The heatmap illustrates the differences in the degree and number of open chromatin regions in peripheral mononuclear cells among individual samples from the HD and KOA groups.


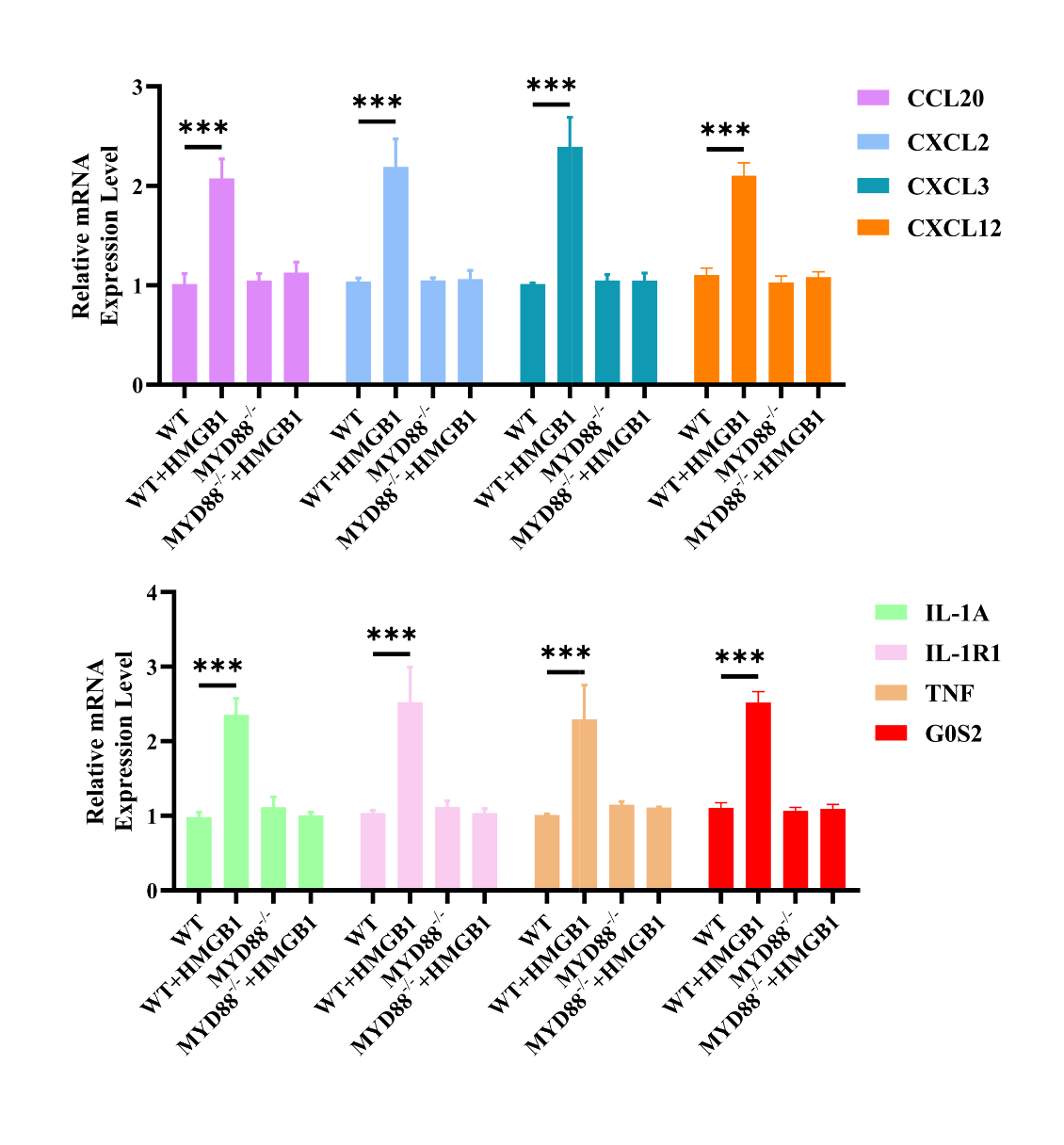


**S-Fig. 6: HMGB1 promotes IRF1 SUMOylation via MyD88 to induce trained immunity in KOA monocytes.**

PCR detection of mRNA expression levels of IL1A, IL1R1, TNF, CXCL12, CXCL3, CXCL2, CCL20, and G0S2 in circulating monocytes from wild-type and MyD88 knockout mice after HMGB1 stimulation. *statistical results were represented by mean ± standard error of the mean (Mean ±* SEM*), *P < 0.05, **P < 0.01,***P < 0.001.*


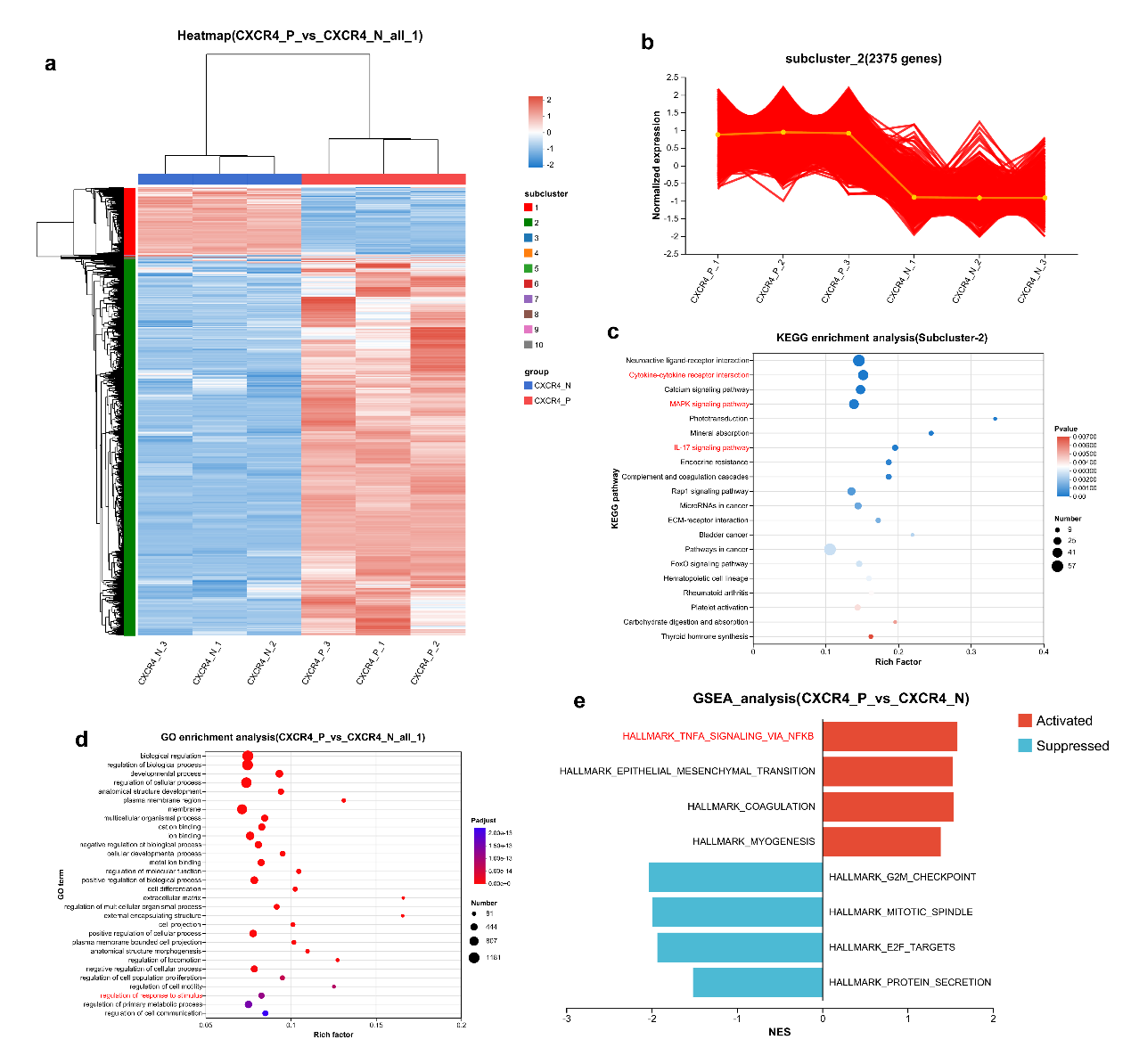


**S-Fig. 8: CXCR4-dependent migration of trained subsets promoted synovial inflammation in KOA.**

(a) Generate a clustered heatmap of differentially expressed genes between CXCR4+ and CXCR4- cells; (b) Plot the expression profile of genes in subcluster 2 identified from the clustering analysis; (c) Perform enrichment analysis of subcluster 2 genes based on the KEGG database; (d) Perform enrichment analysis of subcluster 2 genes based on the GO database; (e) Conduct GSEA analysis of subcluster 2 genes using the HALLMARK database.
