# Supplementary figures and images for: High Mobility Group Protein B1 Promotes Interferon Regulatory Factor 1 SUMOylation to Prime Trained Immunity of Circulating Monocytes and Aggravate the Progressive Synovial Inflammation in Knee Osteoarthritis
Source: Research (Wash D C). 2026 May 14;9:1243. doi: 10.34133/research.1243 (PMC13172585; doi:10.34133/research.1243)

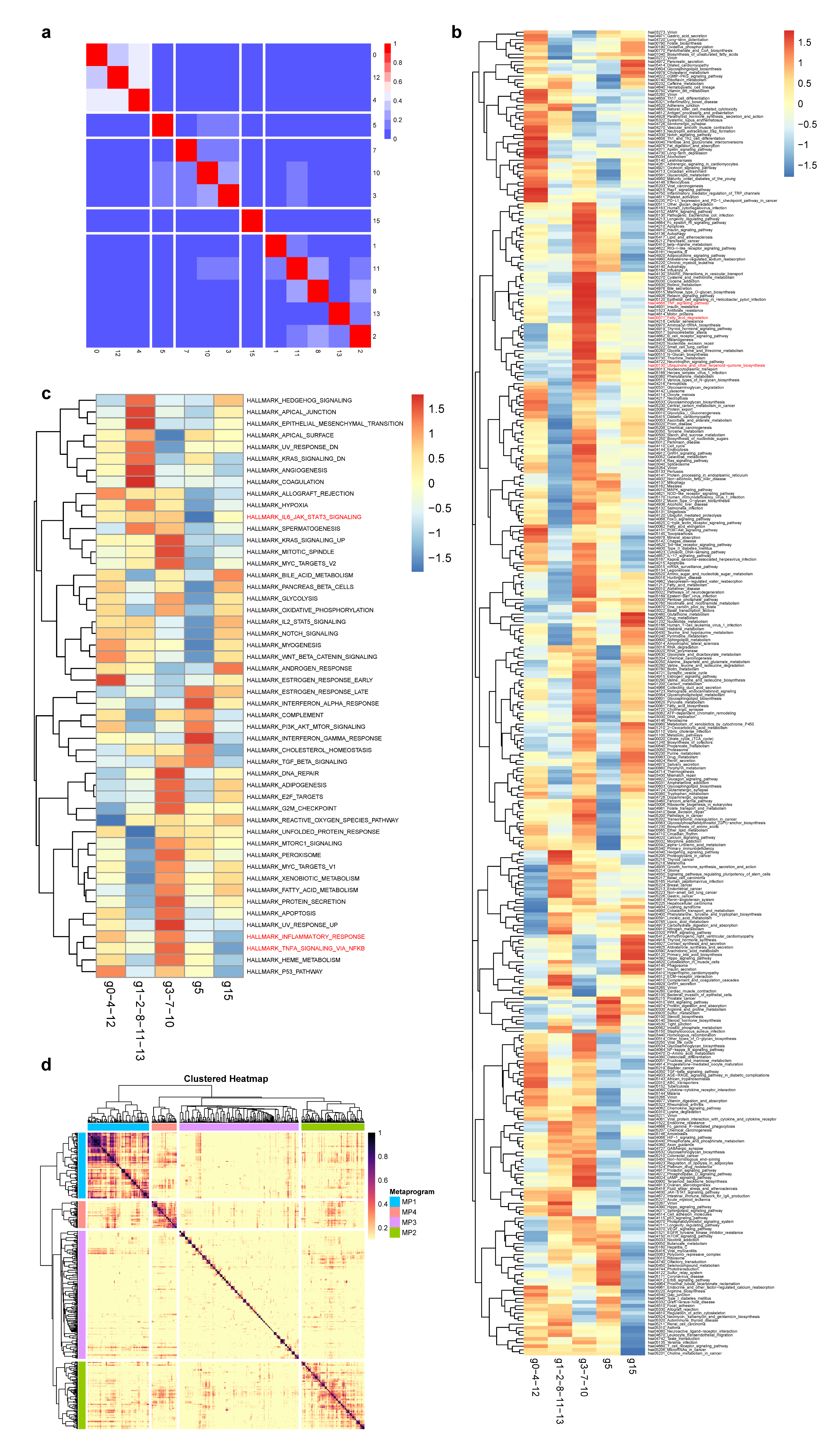

Supplement: Supplementary 1 — Tables S1 and S2 Figs. S1 to S8 [file research.1243.f1.zip › S-figure-1.tif]

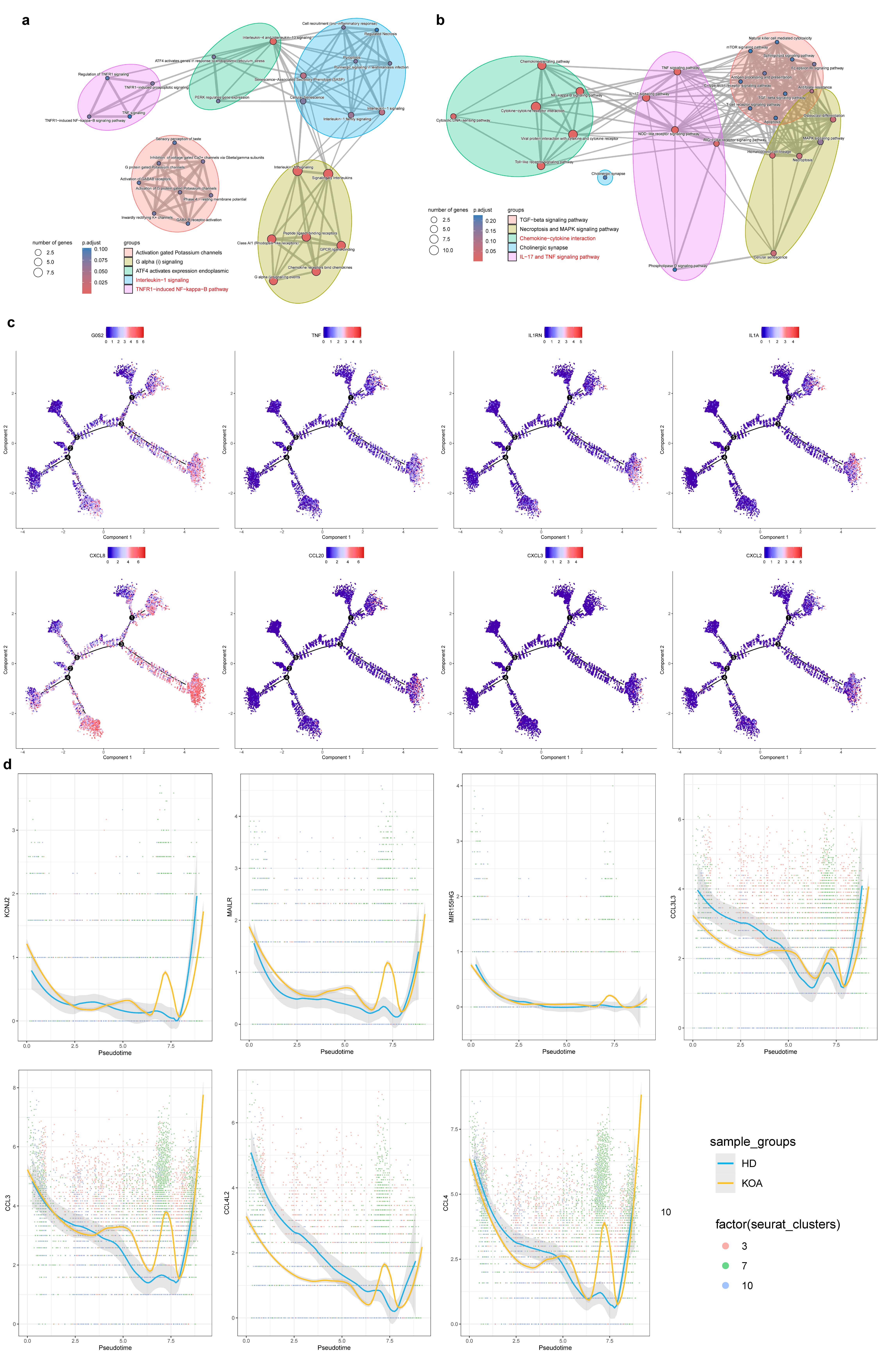

Supplement: Supplementary 1 — Tables S1 and S2 Figs. S1 to S8 [file research.1243.f1.zip › S-figure-2.tif]

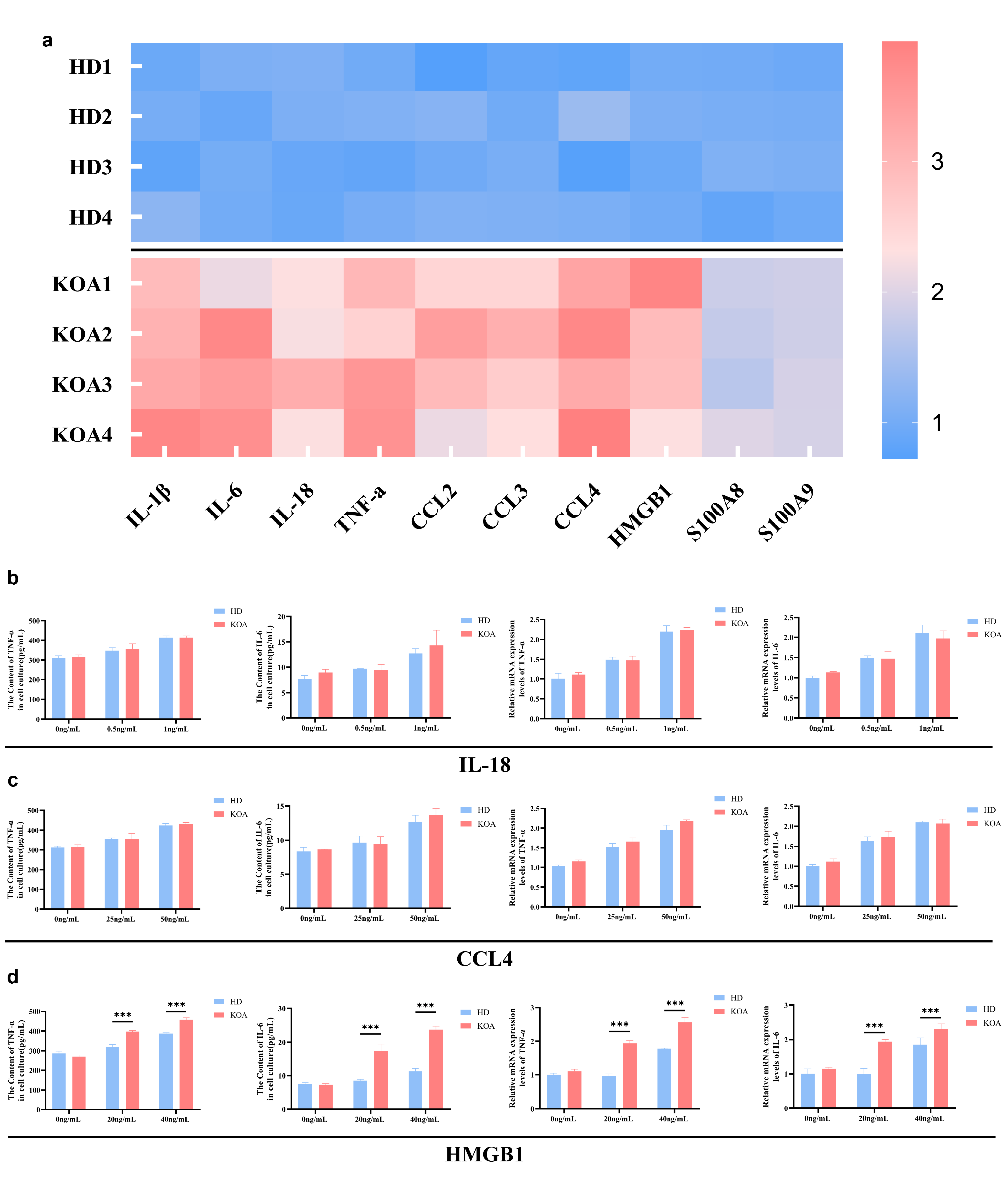

Supplement: Supplementary 1 — Tables S1 and S2 Figs. S1 to S8 [file research.1243.f1.zip › S-figure-3.tif]

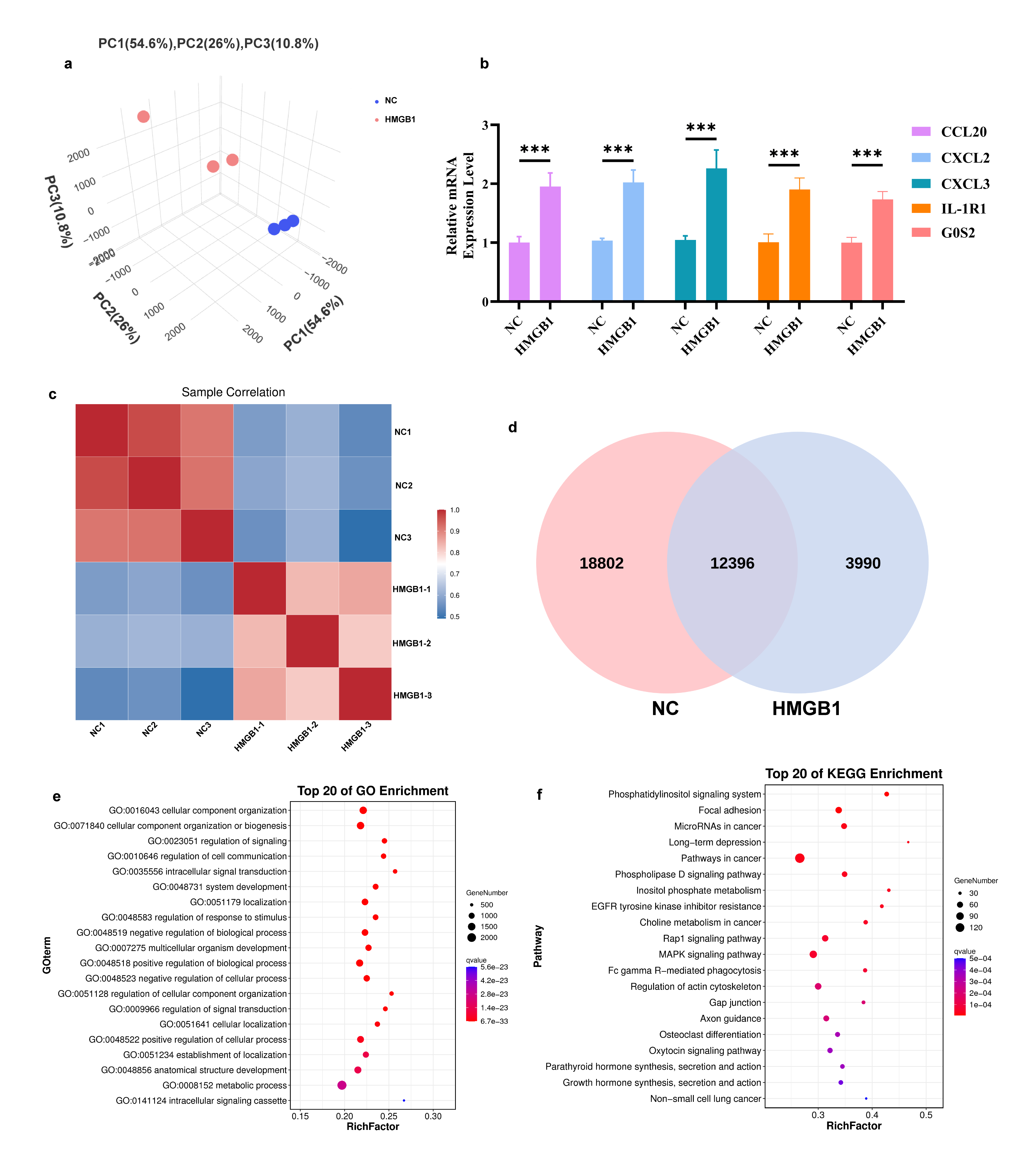

Supplement: Supplementary 1 — Tables S1 and S2 Figs. S1 to S8 [file research.1243.f1.zip › S-figure-4.tif]

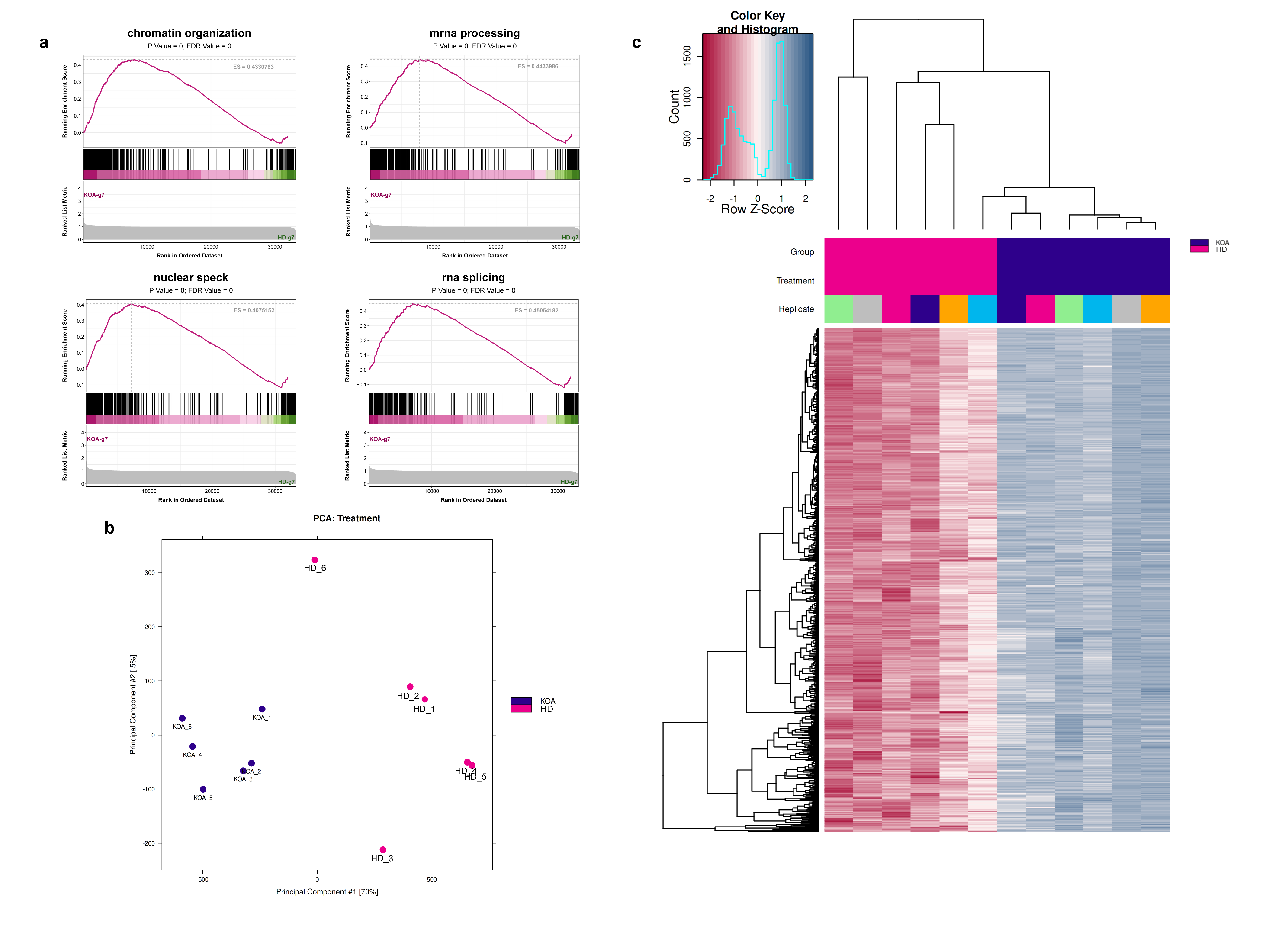

Supplement: Supplementary 1 — Tables S1 and S2 Figs. S1 to S8 [file research.1243.f1.zip › S-figure-5.tif]

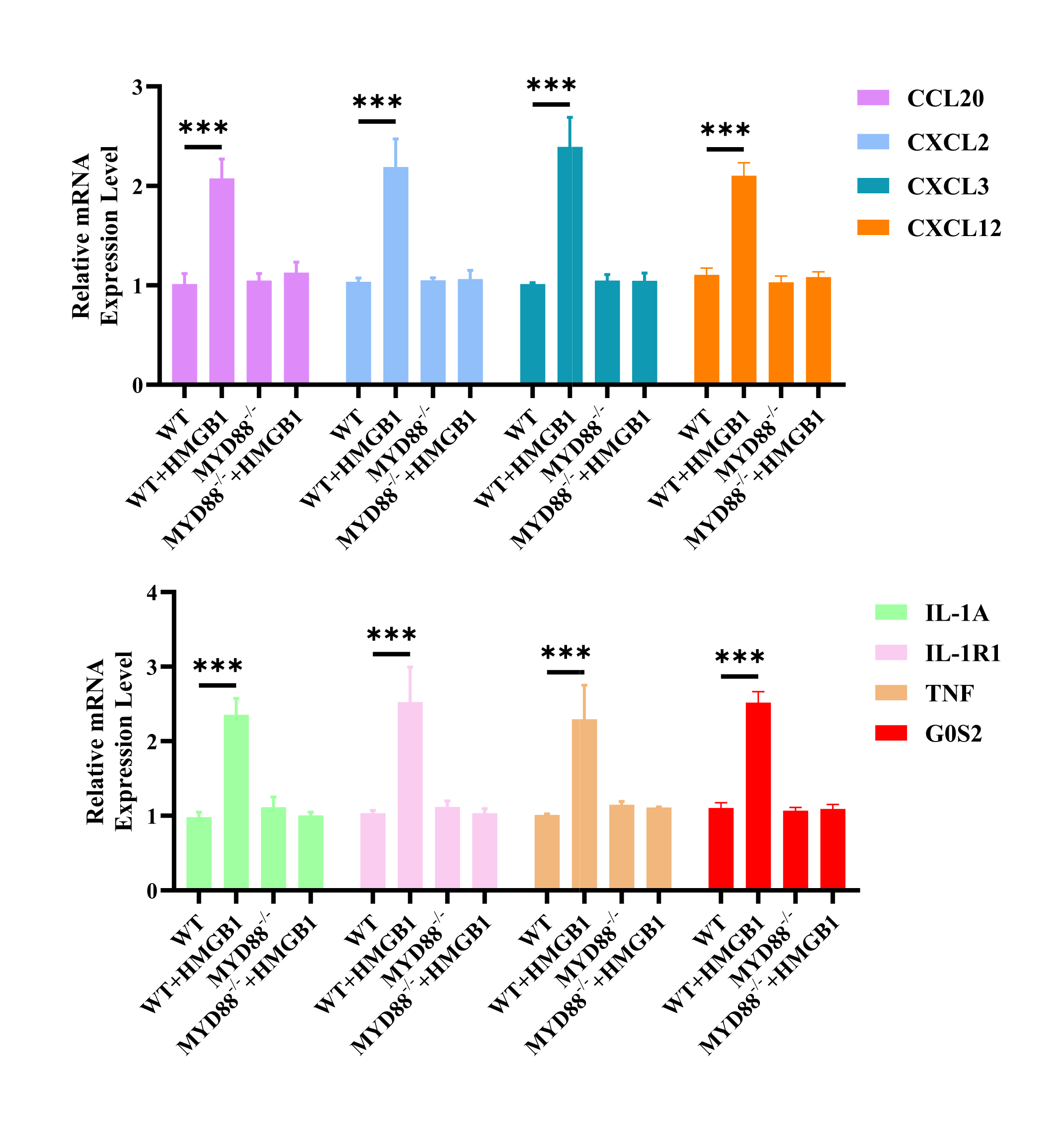

Supplement: Supplementary 1 — Tables S1 and S2 Figs. S1 to S8 [file research.1243.f1.zip › S-figure-6.tif]

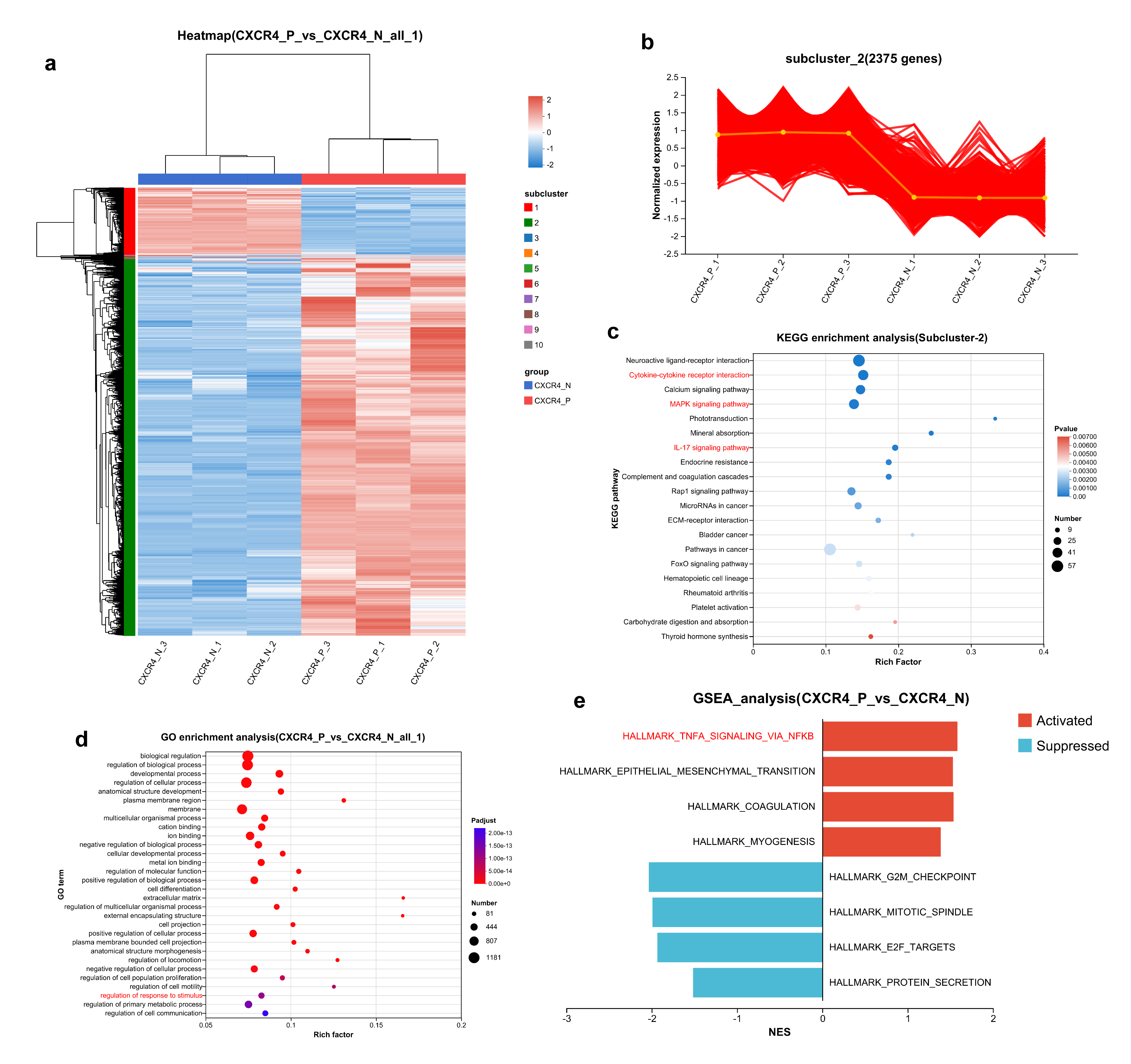

Supplement: Supplementary 1 — Tables S1 and S2 Figs. S1 to S8 [file research.1243.f1.zip › S-figure-8.tif]

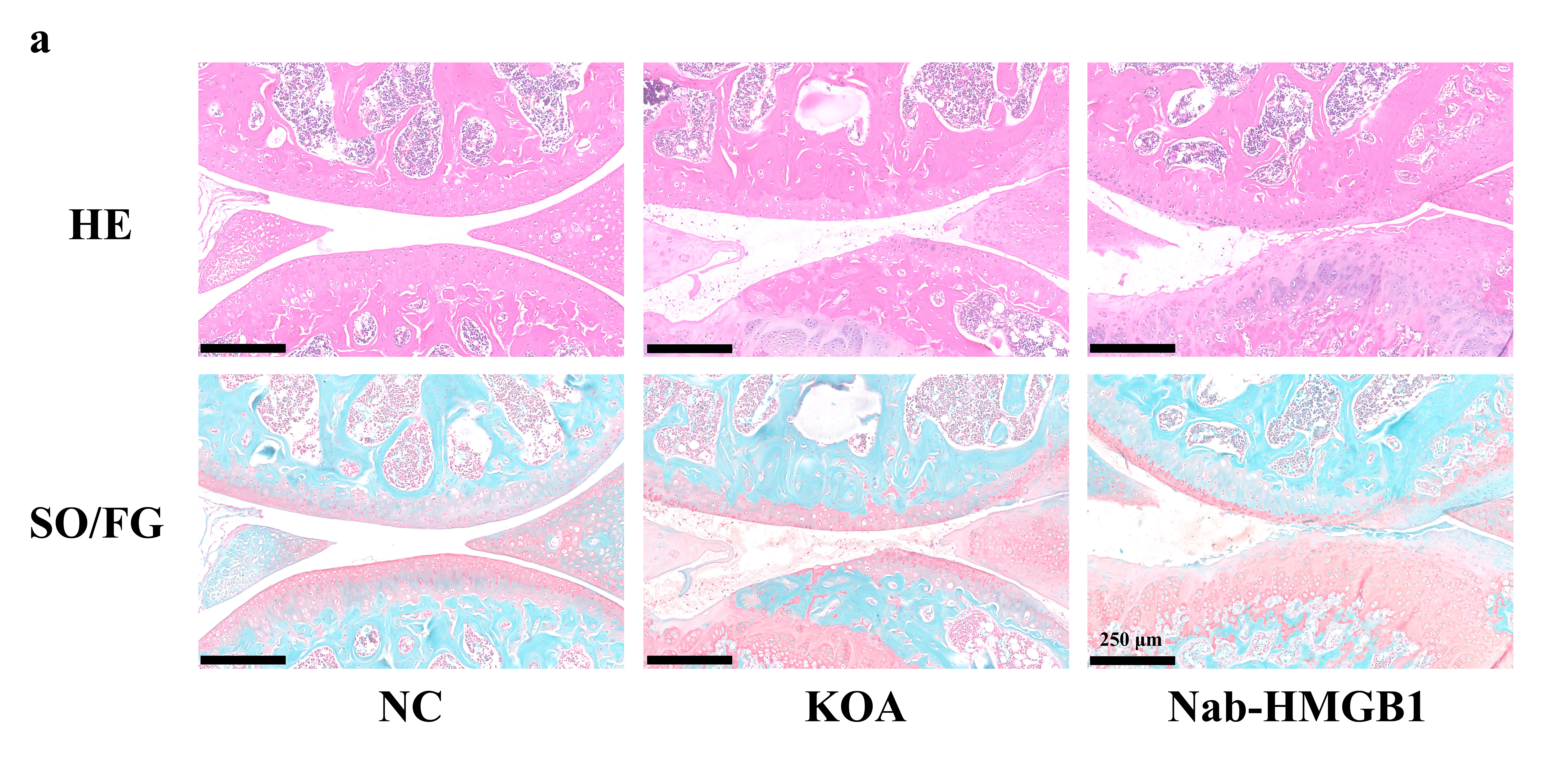

Supplement: Supplementary 1 — Tables S1 and S2 Figs. S1 to S8 [file research.1243.f1.zip › S-figure7.tif]
